# Analysis using size exclusion chromatography of poly(*N*-isopropyl acrylamide) using methanol as an eluent

**DOI:** 10.1016/j.chroma.2017.05.050

**Published:** 2017-07-28

**Authors:** Thomas Swift, Richard Hoskins, Richard Telford, Richard Plenderleith, David Pownall, Stephen Rimmer

**Affiliations:** a School of Chemistry and Biosciences, University of Bradford, Bradford, West Yorkshire, BD7 1DP, United Kingdom; b Polymer and Biomaterials Laboratories, Department of Chemistry, University of Sheffield, Sheffield, S10 2TN, South Yorkshire, United Kingdom

**Keywords:** PNIPAM, Size exclusion chromatography, SEC, DOSY NMR, Methanol, Intrinsic viscosity

## Abstract

Size Exclusion Chromatography is traditionally carried out in either aqueous or non-polar solvents. A system to present molar mass distributions of polymers using methanol as a mobile phase is presented. This is shown to be a suitable system for determining the molar mass distributions poly(*N*-isopropylacrylamide)s (PNIPAM); a polymer class that is often difficult to analyze by size exclusion chromatography. DOSY NMR was used to provide intrinsic viscosity data that was used in conjunction with a viscometric detector to provide absolute calibration. Then the utility of the system was shown by providing the absolute molar mass distributions of dispersed highly branched PNIPAM with biologically functional end groups.

## Highlights

• One of first examples of stable Methanol based SEC system for polymer analysis.
• Universal Calibration determined using DOSY NMR to get intrinsic viscosity.
• Analysis of difficult to characterise branched polyacrylamides.

## 1 Introduction

Polymer molar mass distributions are often obtained by size exclusion chromatography (SEC). SEC must be carried under conditions with no adsorption to the stationary phase and by using stationary phases with pores in the same size range as the size of the polymer coils. This is usually easily achieved for polymers that are soluble in organic solvents using the widely available polystyrene stationary phases. However, SEC of amphiphilic polymers requires careful attention to both eluents and stationary phase to prevent adsorption. For a number of amphiphilic polymers SEC in alcohol eluents would be expected to be not susceptible to adsorption. However, until recently a stationary phase that was suitable for use with eluents such as methanol, ethanol, etc. were not available. New stationary phases are now becoming available, such as the PolarGel series, that are modified to allow use with polar eluents but SEC with alcohol eluents has only recently been reported [1]. In this work we report SEC carried out in methanol and we apply this to the analysis of the molar mass distributions of a range of well studied amphiphilic polymers based on the *N*-isopropyl acrylamide as the main repeat unit.

SEC separates polymers according to their hydrodynamic volume and as such is an extremely solvent sensitive technique. Analysis of samples is restricted by the requirement that the mobile phase must be compatible with both the analyte and the stationary phase. Common mobile phases in SEC are tetrahydrofuran (THF) [2], [3], [4], [5], [6], dimethyl formamide (DMF) [5], [7], [8], [9], [10], [11], [12], [13], [14], Chloroform [4] and water [9] and common calibration standards used include polystyrene [2], [3], [4], [5], [7], polyethylene oxide [8] or polymethyl methacrylate [5], [6], [10], although many variations and alternatives have been reported [15]. However SEC measurements in eluent blends including alcohols have only been reported rarely [16], [17] and there appears to be only one recent report on the use of methanol or ethanol as the sole component of the eluent, in an ultra high pressure SEC system [1]. In some instances preventing adsorption involves modifying organic eluents [18], [19], [20] or using buffered aqueous media [21], [22]. In our experience, the analysis of polyacrylamides often requires changing the eluent if pendant or chain-end functionalities are modified. Some polymers, such as poly(*N*-isopropyl acrylamide) (PNIPAM) are susceptible to adsorption to the stationary phase and often salts, such as tetrabutylammonium bromide (TBAB), must be added to the mobile phase to prevent interaction [12], [23]. The use of salts requires careful control of eluent composition and in some cases salts precipitate and block components of the instrument. A system that does not require the addition of salt would be of great utility and low molar mass alcohols as eluents would be attractive for a number of amphiphilic polymers.

SEC separates the chains on the basis of hydrodynamic volume so that molar mass sensitive detectors using light scattering and/or viscometry are needed to provide absolute molar mass distributions. Viscometric detectors use the Univerisal calibration concept using the relationship shown in Eq. (1), which relates the intrinsic viscosity and molar mass of standard polymers (subscript 1) to the intrinsic viscosity of an unknown (subscript 2).

(1) $${\left[\eta \right]}_{1}M_{1}={\left[\eta \right]}_{2}M_{2}$$

Standards of known [η], obtained in common eluents, and M_n_ and M_w_ are available from commercial sources. However, the use of less common eluents requires determination of [η]; usually using capillary viscometry. Diffusion-ordered NMR spectroscopy (DOSY NMR) could also be used to obtain [η]. DOSY NMR provides similar data to SEC and the distributions of diffusion constants obtained can be transformed to hydrodynamic radius (*R_H_*) or hydrodynamic volume (*V_H_*) distributions, using the Stokes Einstein approach (Eq. (2)). Knowledge of the molar mass distribution allows transformation of the hydrodynamic volume distribution to the distribution of intrinsic viscosities ([η]) using Eq. (3) [24].

(2) $$R_{H}=\frac{k_{B\;}T}{6\pi \eta D}$$

(3) $$[\eta ]=\frac{2.5NV_{H}}{M}$$

Standards with known molar mass and intrinsic viscosity are required for Universal calibration of SEC chromatographs but appropriate standards soluble in methanol (or other alcohols) are not available. Conventionally, intrinsic viscosity of the standard has been obtained from capillary viscometry but it is necessary to relate the average molar mass and average intrinsic viscosity from the same point on the distribution. This is usually achieved by using samples with very narrow dispersity so that the various means converge. However, DOSY provides the facility to obtain average intrinsic viscosities [25] that can be directly related to the average molar mass from the same position on the distribution provided by SEC; both DOSY and SEC provide log distributions with separation provided by the distribution of hydrodynamic volume (V_H_).

With this in mind we have prepared a series of linear PNIPAMs, which were analyzed both by SEC, with viscometric detection with THF as eluent, to get their absolute molar masses, and DOSY NMR in deuterated methanol to determine their intrinsic viscosities in this solvent. The molar mass and intrinsic viscosity were then used to calibrate a methanol based SEC system. This method of analysis provides an improvement over previous published methods of separation and analysis [11] and gives improved separation of the components of the distribution. The use of DOSY NMR to provide the standards with known V_H_ allows for simple calibration of the SEC in terms of the distribution of V_H_ or hydrodynamic radius (R_H_).

## 2 Experimental

### 2.1 Materials

Polymer standards poly(styrene) (PS) and poly(ethylene glycol/ethylene oxide) (PEO) were purchased from Agilent EasiCal/Easivial range. *N*-Isopropyl acrylamide (NIPAM, Acros Scientific 99%), Tris (2-dimethylaminoethyl)amine (Me_6_TREN, Alfa Asar 99% +), methyl 2-chloropropionate (MCP, Aldrich, 97%) and Copper(1) Chloride (CuCl, Aldrich, 99%) were all used as received. *N*,*N*-dimethyl formamide (DMF) and diethyl ether were reagent grade and purchased from Fisher and Sigma Aldrich respectively.

### 2.2 Polymer characterization NMR

Samples were dissolved in a range of deuterated solvents at concentrations of 1 mg ml^−1^. They were analyzed on a Bruker Avance 400 spectrometer, operating at 400 MHz to provide both ^1^H NMR, ^13^C NMR signals and diffusion speed in m^2^ s^−1^. FTIR Solid samples were analyzed using a Perkin Elmer Spectrum 100 with a universal ATR accessory.

### 2.3 Size exclusion chromatography

The moments of the molar mass distributions of especially synthesized PNIPAMs (M_n_ and M_w_) and dispersity (Ð = M_w_/M_n_) of the distributions were obtained using tetrahydrofuran (containing 1% tetrabutylammonium bromide (TBAB)) as eluent. Samples were dissolved in GPC grade tetrahydrofuran (1% TBAB was added to the mobile phase and then the solution was filtered before being used) and injected at a sample concentration of 1 mg ml^−1^ at a flow rate of 1 ml min^−1^ through two PL gel mixed-B columns. Sample elution was measured using Agilent refractive index and viscometric (Agilent 1260 Infinity Detector Suite) detectors (at 30 °C) to give the absolute molar mass distributions (see electronic supporting information for full calibration details).

### 2.4 Size exclusion chromatography with methanol as eluent

To condition the columns, pre-filtered solvent was flushed through two Agilent Polargel columns for 24 h at 0.5 ml min^−1^ before the solvent reservoir was set to recirculate. The isocratic pump pressure was approximately 4000 kPa, with a ripple of 0.51, and the viscometer internal pressure (IP) was set at 44.5 kPa. Samples were prepared in pre-filtered Methanol (1 mg ml^−1^) and injected through two Agilent Polargel columns (1 ml min^−1^) maintained at 30 °C. This was then fed through Agilent UV (254 nm), refractive index and viscometric (Agilent 1260 Infinity Detector Suite) detectors each maintained at 30 °C. Poly(ethylene oxide) polymer standards provided by Agilent were found to be partially retained by the column (see electronic supporting information) and so PNIPAM standards were prepared using atom-transfer radical polymerization (ATRP). The column set had a hold up volume of 33.2 cm^3^ and an internal pore volume of 19.74 cm^3^ determined from the retention time of a non-retained molecule, toluene, and fully excluded high molar mass PNIPAM (see electronic supporting information).

### 2.5 Synthesis of linear PNIPAM standards by ATRP

Seven linear PNIPAM polymers were prepared using ATRP where the ratio of monomer:iMCP:CuCl:Me_6_TREN was varied to give polymers of differing molar masses. For a polymer with ratio 50:1:1: 1. *N*-Isopropylacrylamide (NIPAM) (2 g) was dissolved in a solution of DMF (4.5 ml) and water (3 ml) and purged with nitrogen for 30 min. A stock solution of CuCl/Me_6_TREN was prepared consisting of CuCl (0.15 g) and Me_6_TREN (0.33 g) in degassed water (6 ml) and then degassed by purging with nitrogen. 1.5 ml of the stock solution was added to the NIPAM together with MCP (50 mg). The resulting solution was stirred under nitrogen for 2 h and the polymer obtained by precipitation twice in diethyl ether. The excess copper was removed by dissolving the polymer in THF (10 mg ml^−1^) and passing through an alumina column before it was reprecipitated in diethyl ether for a final time. The polymers were analyzed by FTIR and THF SEC. ^1^H NMR (D_2_O) 1.04 (CH_3_)_2_, 1.4–2 (CHCH_2_), 3.84 (CH) and 7.3 (NH). Additional polymers were prepared using FeCl_2_ opposed to CuCl (see Supporting information).

## 3 Results and discussion

### 3.1 Preparation of methanol size exclusion chromatography technique with PNIPAM calibration standards

#### 3.1.1 Synthesis of linear polymers by atom transfer radical polymerization (ATRP)

**Fig. 1:**
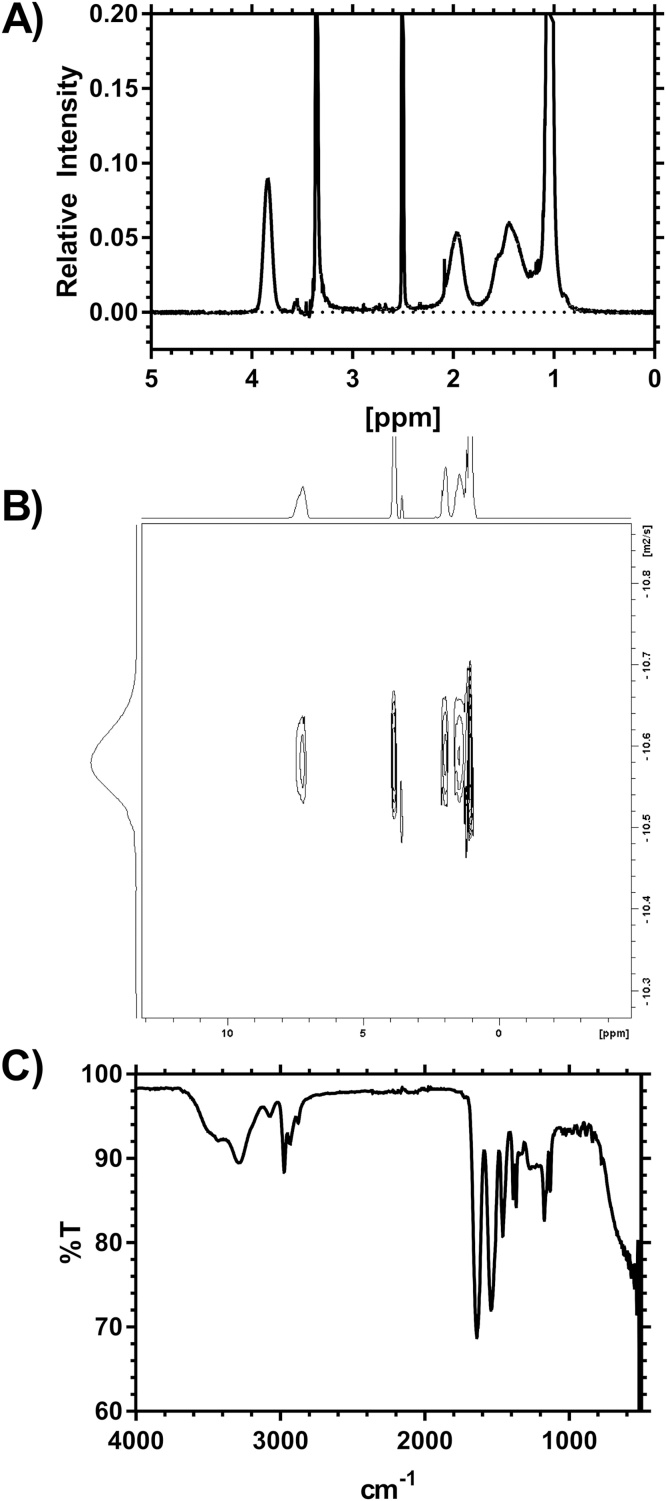
^1^H NMR (A), ^1^H DOSY NMR (in D_2_O) (B), and FTIR (C) spectra of PNIPAM 9. Expanded spectra and additional information are shown in the electronic Supporting information.

It was found that traditional aqueous soluble polymer standards, polyethylene oxide (PEO) although soluble in methanol were partially retained by interactions with the column giving an unusable calibration plot. This is fully documented in the electronic supporting information. Given these issues with traditional PEO standards the system was calibrated using especially synthesized linear PNIPAM materials. Seven polymers were produced using a protocol outlined by Masci et al. [5]. The properties of these polymers are shown in Table 1 and they were characterized by NMR and FTIR (Fig. 1) (further information is shown in the electronic supporting information). In our hands these polymerizations produced polymers that showed increasing dispersity with increasing molar mass, following the reduction in molar ratio of ATRP initiator/catalyst to monomer in the reaction feed.

**Table 1:** Properties of Linear PNIPAM standards.

	M: I^a^	M_p(THF)_^b^	M_n(THF)_^b^	Ð^b^	R_Hp(D2O)_^c^	R_Hp(MeOD)_^c^	R_Hp(THF)_^c^
1	9600:1	1405.50	1101.36	2.38	13.52	13.32	13.12
2	800:1	240.05	128.63	2.29	8.07	7.32	7.78
3	600:1	238.50	116.92	2.31	6.76	6.50	6.56
4	400:1	122.28	55.08	2.12	5.51	4.87	5.06
5	200:1	45.00	41.06	2.01	3.56	3.36	3.25
6	100:1	39.00	34.81	2.24	3.39	3.02	3.01
7	100:1	28.00	22.61	2.34	3.16	2.63	2.73
8	100:1	18.50	18.73	2.08	2.57	2.29	2.31
9	50:1	14.00	15.98	1.81	2.24	2.09	2.11

a Monomer(PNIPAM):Initiator(MCP) concentration. Iniator:CuCl:Me_6_TREN ratio constant at 1:1:1.

b Polymer molar mass, M_n_ (kg mol^−1^), and dispersity analyzed using size exclusion chromatography in THF (1 wt%TBAB,).

c R_Hp_, peak hydrodynamic radius (nm) analyzed using DOSY NMR measurements.

The DOSY method (at 25 °C) was used to compare the hydrodynamic radii (R_H_) of the linear PNIPAMs in water, methanol and THF. The peak values (R_Hp_) of the distributions are tabulated in Table 1 (each point showing the mean from five measurement with an average standard deviation <0.1 nm, (see Supporting information for full details). The hydrodynamic radii were consistently higher in D_2_O (paired *t*-test p < 0.01) than in either THF or methanol. However, there was no significant difference observed between the R_Hp_ obtained in methanol and THF (paired *t*-test p = 0.3713).

The data indicated greater solvation of these linear PNIPAMs in water at 25 °C but the high viscosity of water usually requires the use of elevated temperatures in SEC, which given the LCST behavior of PNIPAM, means that water is not a suitable medium. Importantly the equivalence of R_Hp_ in THF or methanol allows for the assumption that the peak molar mass average (M_p_) obtained from a SEC system using THF with conventional (polystyrene) columns could be used with [η] derived from DOSY in methanol to provide a universal calibration of a SEC system using methanol as the eluent with suitable columns.

#### 3.1.2 Calibration of PolarGel stationary GPC columns

PolarGel stationary phases are compatible with THF so that SEC of PNIPAMs 2, 4 and 9 was attempted using PolarGel columns with THF as the mobile phase and it was found that the samples eluted. However the refractive index response was both small and subject to significant baseline drift (see Electronic Supporting information). This indicated that using these columns with THF as eluent was not reasonable because the separation process would be expected to be a mixture of adsorption and size exclusion. Data that are not subject to adsorption were obtained using methanol as an eluent. However as no dilute solution viscosity data, in methanol, was available it was necessary to determine intrinsic viscosities before Polymers 1–9 could be used to provide a universal calibration.

The R_Hp_ data shown in Table 1 were obtained from the diffusion constants obtained from DOSY NMR by using Eq. (2). These data were then used to provided the peak average of the intrinsic viscosity ([η]_p_) by application of Eq. (3).

**Fig. 2:**
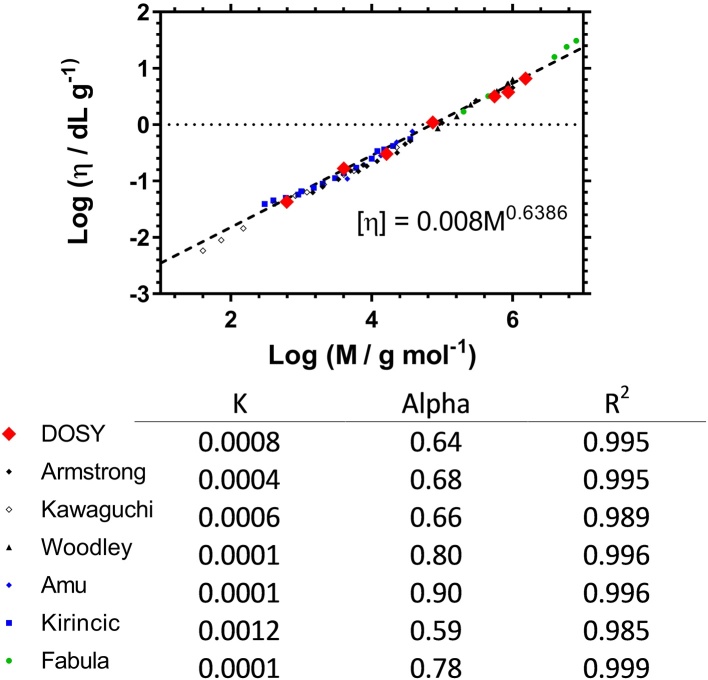
MHS Plot for PEO of varying molar masses calculated from NMR diffusion measurements (♦) compared to measurements from previous studies (dilute solution viscometry) with calculated K and α [24], [26], [27], [28], [29], [30], [31].

In order to validate the DOSY method (using Eqs. (1) and (2)) [η]_p_ was obtained in D_2_O for a set of narrow dispersity polyethylene glycol (PEG) standards of known molar mass and the values were compared to the [η]_w_ in water obtained from the literature [24], [26], [27], [28], [29], [30], [31]). The data and the literature values are presented in Fig. 2 by plotting log[η] versus logM (see also Supporting data) following the Mark-Houwink-Sakarada (MHS) equation (Eq. (4)). The MHS equation is usually used to relate empirically derived values of the intrinsic viscosity to the viscosity average molar mass (M_v_) and here we use this approach to compare the values of log [η] derived from DOSY to those derived from dilute solution viscometry.

(4) $$[\eta ]=K{M_{V}}^{\alpha }$$

Fig. 2 shows that [η] obtained by the DOSY technique was the same within experimental error as the literature reported values; linear regression showed R^2^ = 0.993 for all of the combined data. An important part of the DOSY method for obtaining [η] was the realization (see electronic Supporting information) that the viscosity of the medium (η in Eq (1)) could be obtained by using Eq. (5).

(5) $$D_{1}\eta_{1}=D_{2}\eta_{2}$$

Where the subscripts refer to different concentrations of polymer and the diffusion constants, D, are the self-diffusion constants of the pure solvent and the solvent in a mixture of polymer and solvent. Thus, a knowledge of the self-diffusion constant of the pure solvent (D_1_) and the viscosity of pure solvent (η_1_) allows the viscosity of the combined medium (polymer + solvent, η_2_) required in Eq. (1) to be obtained from D_2_. This method validates the use of the Stokes-Einstein equation (Eq. (2)) to give accurate hydrodynamic radii (*R_Hp_*) from DOSY measurements and in turn gives accurate intrinsic viscosities of the polymer in solution (Eq (3)). It is worth noting that although the viscosity of deuterated solvents can differ from ^1^H isotope solvents [32], the intrinsic viscosity of polymers within deuterated solvents are equivalent [33].

The use of DOSY NMR to obtain [η] from the peak of a diffusion constant distribution is independent of the molar mass distribution if the molar mass of the standard is taken from the peak of the SEC data (M_p_) because both techniques (SEC and DOSY NMR) provide equivalent weight distributions of log R_H_. Therefore, PEG standards were used to obtain the plot shown in Fig. 2 and PNIPAM standards were prepared using ATRP.

**Fig. 3:**
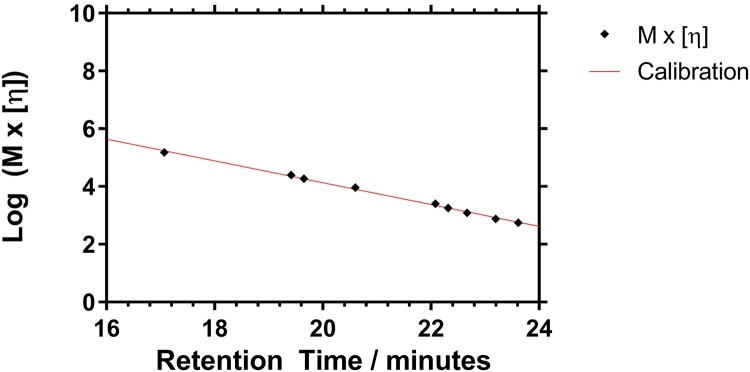
Calibration of Agilent PolarGel columns with linear PNIPAM polymers.

The THF SEC system was used to provide M_p_ and the DOSY data provided the corresponding [η]_p_ for each PNIPAM standard in methanol. For calibration of the methanol system each sample was analyzed at a concentration of 1 mg ml^−1^ with a 100 μl injection loop. The peak retention time of these polymers was then used to calibrate the system to afford both a SEC calibration plot (see Supporting information) and a Universal calibration (Fig. 3) by plotting log(M[η]) versus retention time [34].

Fig. 3 shows that the data provided a linear Universal calibration plot, which was an indication that these PNIPAM standards were fractionated by size exclusion with minimal adsorption of the polymer to the column. Full data is shown in the electronic Supporting information.

To validate the technique a PNIPAM standard was purchased from Sigma Aldrich (quoted M_V_ 63,200, Ð 2.13) and was analyzed by Methanol SEC. The SEC indicated that the M_V_ was 66,000 g mol^−1^ and Ð = 1.96. Therefore, the data derived from methanol based SEC are in good agreement with the previously obtained values for this standard.

**Fig. 4:**
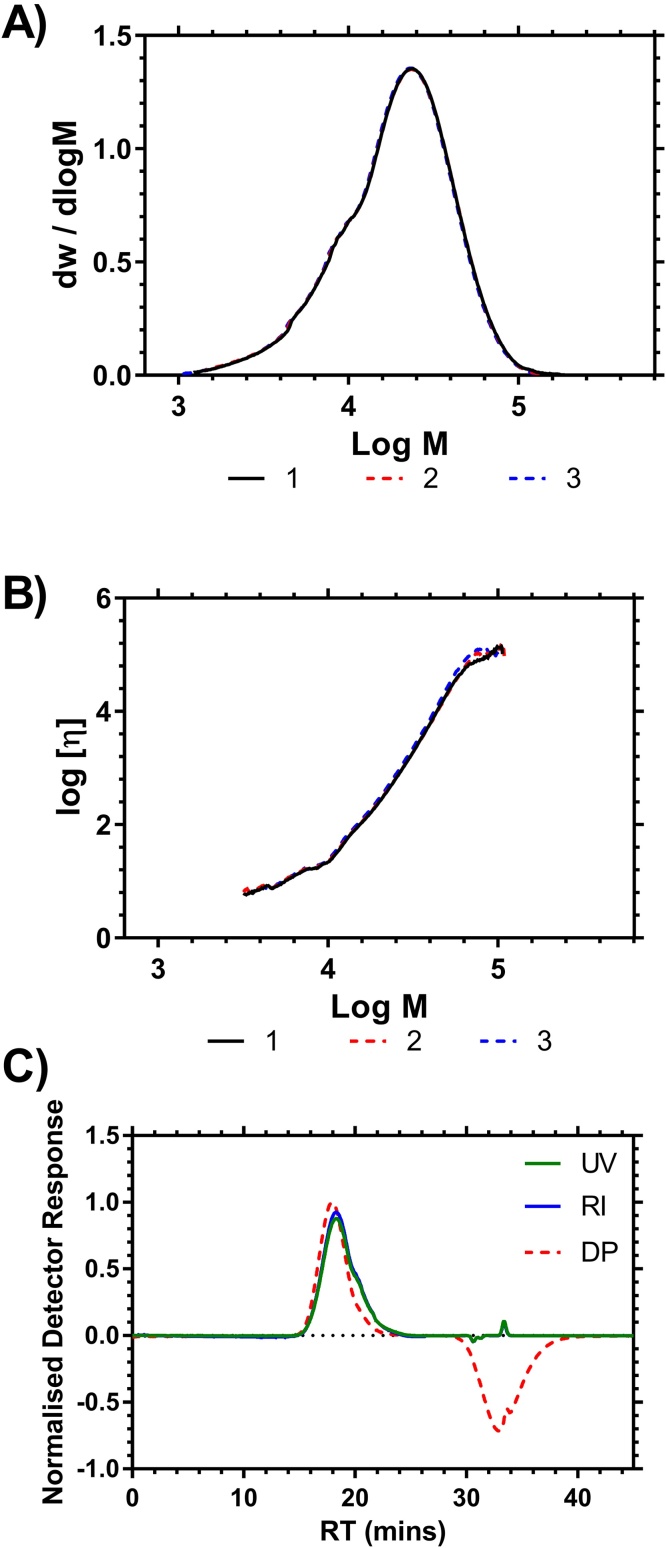
Molar mass distribution (A) and MHS plot (B) and raw detector responses (C) of linear PNIPAM **8** over three repeat measurements of SEC Methanol as the eluent.

Another linear PNIPAM Polymer prepared by Fe ATRP (see Electronic Supporting Information) was measured nine times over two separate days to test the reproducibility of the system. Each repeat measurement of this sample gave essentially indistinguishable molar mass distributions and equivalent MHS plots which were superimposable (Fig. 4). The polymer produced in this way was more disperse than the standards produced using Cu ATRP. The moments of molar mass were determined and are shown in Table 2. The coefficients of variation calculated for each sample illustrate the excellent reproducibility of the system and for each parameter the variation is less than 2% of the mean.

**Table 2:** Molar mass moments of linear PNIPAM prepared using Fe ATRP over nine repeat measurements using SEC with methanol as eluent. S.D. = standard deviation; C.V. = coefficient of variation.

Repeat	M_N_^a^	M_w_^a^	M_z_^a^	Ð	k/dl g^−1^	α
1.1	18.74	28.75	37.56	1.53	0.00408	0.66
1.2	18.53	27.76	37.05	1.50	0.00378	0.67
1.3	18.07	27.61	37.32	1.53	0.00398	0.67
2.1	17.85	27.93	37.81	1.56	0.00412	0.66
2.2	18.81	28.26	37.76	1.50	0.00398	0.67
2.3	18.29	28.5	37.75	1.56	0.00384	0.66
3.1	18.41	27.31	36.74	1.48	0.003999	0.67
3.2	18.55	28.12	37.57	1.52	0.00391	0.66
3.3	18.55	27.53	37.24	1.48	0.00377	0.66
Mean	**18.42**	**27.97**	**37.42**	**1.48**	**0.00394**	**0.664**
S.D.	0.31	0.47	0.36	0.02	0.00012	0.005
C.V	0.017	0.017	0.010	0.014	0.031	0.008

a Polymer molar mass in kg mol^−1^.

The exponent of the MHS equation (Eq. (3)), α, is obtained from a plot of log M against log [η] and these values can be used to provide an indication of the solvation or architecture of the polymer in solution. α = 0.5 for a linear polymer indicates that the polymer is at the point of incipient precipitation; the theta point. Solvated polymer chains in random coil conformations typically have α ≈ 0.7 and larger values up to 2 are indicative of rigid rod type structures. α < 0.5 cannot be achieved for linear polymers and indicates more compact architectures, typically branched architectures. Table 2 shows that α = 0.67 (std. dev = 0.005) indicating that this linear polymer, as expected, is solvated and that the polymer had a random coil conformation in methanol.

### 3.2 Application of methanol GPC as tool for studying highly branched (HB) PNIPAM polymers

**Fig. 5:**
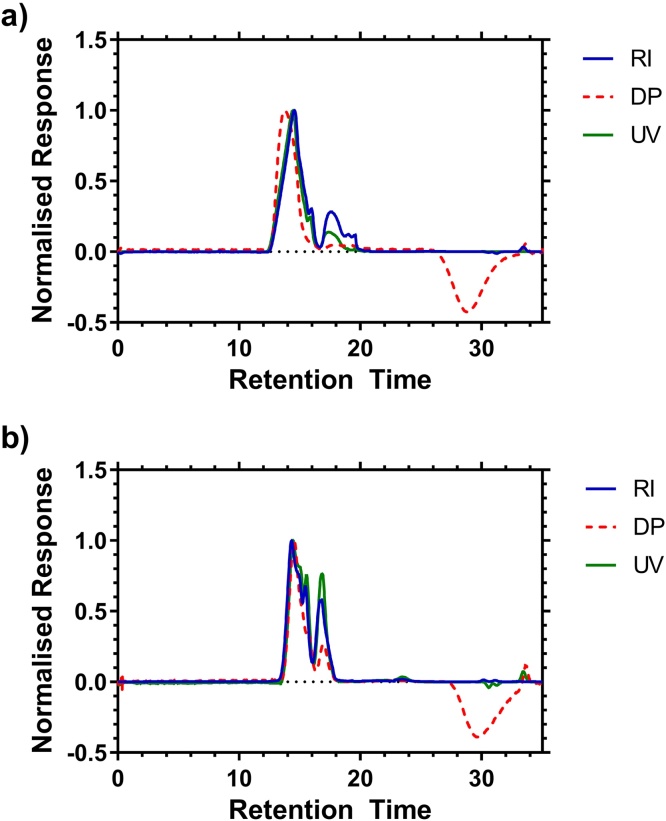
Analysis of HB-PNIPAM. SEC chromatograms, derived from differential refractive index, ultra-violet light absorbance or differential viscometry detectors. HB-PNIPAM with feed ratio of NIPAM:VBP a) 15:1 b) 85:1.

HB-PNIPAM has been prepared previously for a number applications [11], [13], [14] and we considered that methanol as an eluent would provide a general system for the analysis of these and other complex amphiphilic polymers. On the other hand, in our experience the alternative eluents, DMF, THF + tetrabutylammonium bromide or aqueous systems are often useful for only a narrow range of compositions. Thus, HB-PNIPAMs were prepared by copolymerization of NIPAM with 4-vinylbenzyl pyrrolecarbodithioate (VBP) (previously described by Plenderleith et al. [11]) were analyzed using the methanol SEC system and it was found that these polymers had bimodal molar mass distributions (Fig. 5). This itself is not unexpected and has previously been reported, however size exclusion chromatography in DMF did not fully resolve the two peaks into separate components. Furthermore the size distribution of the two components changed depending on the degree of branching of the polymer, with the smaller component of the highly branched (15:1) peak giving almost no response in differential pressure whilst in less branched samples the RI, DP and UV detector responses were almost superimposable.

**Fig. 6:**
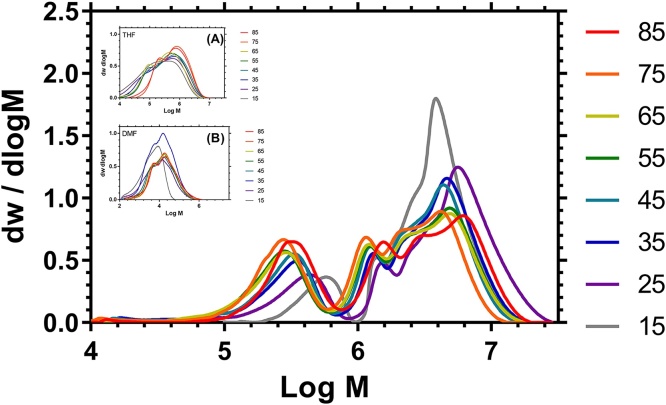
Molar Mass Distributions of HB-PNIPAMs (PNIPAM: VBP feed molar ratio shown to the right) determined by Methanol GPC. INSET: Distributions obtained using polystyrene columns with: a. THF as eluent (see Supporting information) or b. previously published distributions using DMF as eluent [11].

The molar mass averages of these polymers were calculated using the Universal calibration. The two bi-modal peaks were treated as one polymer distribution and M_N_, M_W_ and M_Z_ values were calculated. The Ð of a polymer molar mass distribution is usually described using a single parameter, M_W_/M_N_. Given the bimodal nature of these distributions we consider that a better description of the data can be provided using two dispersities (Ð_W_ = M_W_/M_N_, Ð_Z_ = M_Z_/M_W_). Ð_W_ was highly influenced by the lower molar mass tail whereas Ð_Z_ shows variation predominantly reflecting the broadness of the larger molar mass component. The full sets of data are shown in Table 3 and Fig. 6. The same samples were analyzed using THF with added TBAB and crosslinked polystyrene as the stationary phase as shown in Fig. 6 inset. The data clearly show the improved separation obtained with methanol/Polargel system (see supplementary information for further details).

**Table 3:** Molar Mass Moments of HB-PNIPAM obtained from Methanol SEC.

NIPAM: VBP^a^	M_N_^b^	M_W_^b^	M_Z_^b^	Ð_W_	Ð_Z_	α_1_^c^	α_2_^c^
15	1834.7	3766.0	5338.3	2.05	1.42	0.493	0.748
25	1133.9	5022.1	8631.1	4.43	1.71	0.328	0.785
35	555.4	3357.2	5885.6	6.04	1.75	0.463	0.715
45	532.3	2909.4	5144.2	5.46	1.77	0.220	0.691
55	438.8	2888.8	5552.3	6.58	1.92	0.307	0.518
65	245.6	3452.7	6940.8	14.06	2.01	0.234	0.531
75	412.5	2265.5	4298.0	5.49	1.90	0.209	0.491
85	624.6	3352.1	6548.3	5.37	1.95	0.303	0.490

a Molar polymerization feeds, NIPAM:VBP.

b In kg mol^−1^.

c Molar mass range Log 5–6 (α_1_) and log 6–7 (α_2_).

**Fig. 7:**
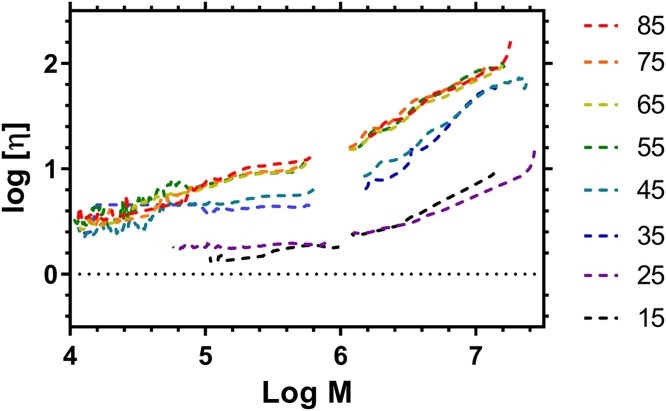
Plots of Log[η] versus logM for HB-PNIPAM (PNIPAM:VBP molar feed ratio shown to the right) determined by Methanol SEC.

The separation in SEC occurs on the basis of hydrodynamic volume and for branched polymers a slice of constant hydrodynamic radius (dR_H_) contains local dispersity in both degree of branching and molar mass at constant R_H_ [35]. Not withstanding this comment, for highly branched polymers the local dispersity in a SEC chromatogram is thought to be negligible [35]. A plot of log [η]_p_ versus log M_N_ should be linear in dilute solution if the degree of branching and therefore α (from Eq. (4)) is constant. Fig. 7 shows these plots for the data provided in Fig. 6. The data show that across the molar mass distribution the MHS plots deviate from linearity and from inspection there appear to be two regions with different values of α, above and below 10^6^ g mol^−1^. This indicates a change in the degree of branching (changing α) as the average molar mass changes.

Deconvolution of the two peaks of the two main regions of the data shown in Fig. 6 can be used to calculate two separate values for α (obtained between: 5 < log M < 6 and 6 < log M < 7) and these separate values are shown in Table 3. The results showed that the lower molar mass fraction had a higher degree of branching (α_1_ < α_2_). As expected for highly branched polymers the α_1_ values were generally less than the theoretical limit for linear polymers in solution (α < 0.5). However, the higher molar mass fraction had α_2_ that varied from <0.5 to 0.95.

**Fig. 8:**
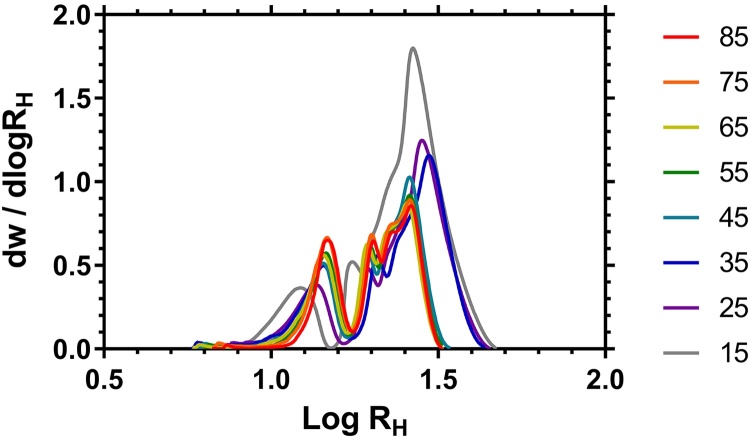
R_H_ Distributions of HB PNIPAM (PNIPAM:VBP molar feed ratio shown to the right) determined by Methanol SEC.

It is likely that for many applications reporting the size of branched polymers is equally as important as the molar mass distributions. Therefore, in Fig. 8 the data in Fig. 5 were transformed into distributions of R_H_ and the peak, number average and weight average radii are shown in Table 4, alongside similar data generated using DOSY NMR (T = 25 °C) of these samples in methanol.

**Table 4:** R_H_ of HB-PNIPAM polymers from Methanol SEC.

SEC			DOSY			
NIPAM: VBP^a^	R_Hn_	R_Hw_	−logD (S)^b^	-logD (P)^b^	R_Hn_	R_Hw_
MeOD	–	–	9.087	–	–	–
15:1	19.7	26.38	8.616	10.224	23.6	24.5
25:1	17.89	27.40	8.648	10.243	23.6	24.5
35:1	16.29	27.08	8.636	10.208	22.9	24.0
45:1	15.71	24.89	8.625	10.182	20.6	21.3
55:1	16.02	24.04	8.641	10.207	20.4	20.9
65:1	15.33	23.39	8.663	10.215	19.8	20.4
75:1	16.05	23.41	8.636	10.212	19.9	20.6
85:1	16.93	23.89	8.639	10.211	20.4	21.0

a Molar reaction feed.

b Diffusion of solvent (S) and polymer (P) ^1^H proton peaks.

The number averages (R_Hn_) determined from the methanol SEC are similar, and largely follow the trend of the peak found in the DOSY NMR experiment (R_Hp_) when these samples were analyzed in deuterated methanol. However although these techniques provide comparable data regarding average size, the SEC experiment gives much greater resolution of the distribution of the radii. The DOSY data showed a shoulder that could be deconvoluted to two separate peaks but the resolution seen on the SEC could not be achieved (see Supporting Information). With further development of the DOSY method however greater increases in resolution may be possible but for the time being SEC remains the most accurate way of determining the full molar mass distributions of highly disperse samples.

**Fig. 9:**
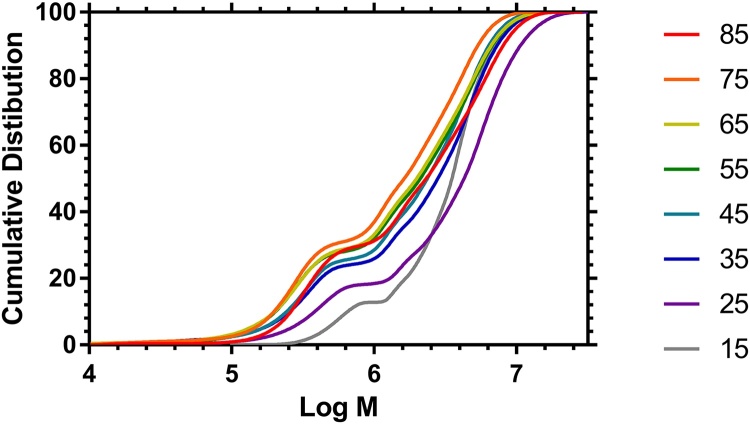
Cumulative Molar Mass Distributions of HB PNIPAM Polymers (PNIPAM:VBP molar feed ratio shown to the right) determined by Methanol SEC.

Robust statistical comparisons of molar mass distributions is difficult and is a still unresolved problem. Branched polymers typically provide non-parametric molar mass distributions and the usual procedure of citing only a single dispersity and the moments of the distribution go only part of the way to providing a useful description [36]. An alternative is to provide the cumulative molar mass distributions and these are shown in Fig. 9. Comparison of cumulative distributions obtained using methanol as the eluent show a clear difference between the distributions obtained at feed ratios of 15:1 and 25:1 and the other feed ratios. A full explanation of these differences will require simulation of the kinetics but at the current time all of the parameters (e.g. the rates of transfer to the dithioate) are not available. A qualitative explanation of the data would assume that the results reflect changes in the dominance of the various processes during polymerization.

### 3.3 Application of methanol SEC as a tool for studying HB-PNIPAM with functionality for binding to bacteria

**Fig. 10:**
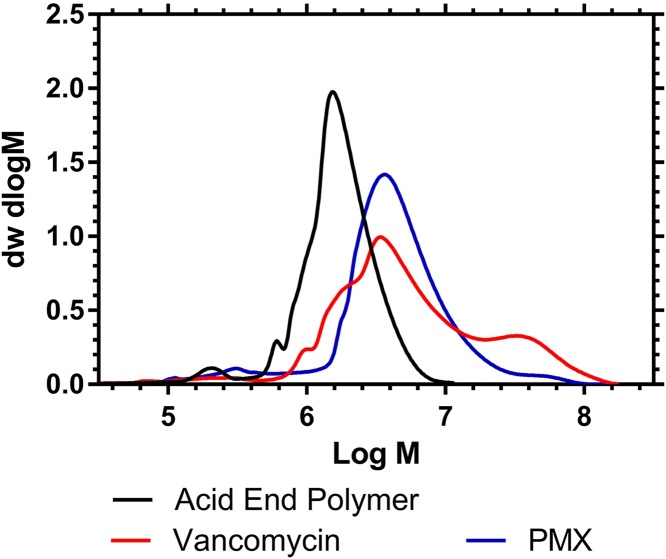
Molar Mass Distributions of chain end functionalized HB-PNIPAMs, obtained from HB-PNIPAM modified polymers with initial feed ratios of 25:1.

These HB-PNIPAMs can be functionalized with antimicrobial end groups to give materials that respond to the presence of bacteria. However, chain end functionalization affects their solubility in traditional SEC solvents [23], [37], [38]. The advent of a methanol based system means for the first time it will be possible to disclose the molar mass of these materials before and after chain-end modification. Two previously published systems are vancomycin functional polymers for targeting gram positive bacteria [37] and polymyxin functionalized polymers for targeting gram negative bacteria [38]. In this work we have re-examined polymers from these previous studies (see Supporting information) and the molar mass distributions of these materials are shown below in Fig. 10.

These data show that conversion of the carboxylic acid chain end functional polymer to antimicrobial functional materials, and subsequent purification (see electronic Supporting information), removed the relatively low molar mass (10^5^–10^6^ g mol^−1^) fraction. This shows that these polymers, which have been shown to be stimuli-responsive to bacteria in solution and to be useful for aggregation of bacteria [23], [37], [38], had broad molar mass distributions and that molar mass distributions changed following modification and purification (ultra-filtration and precipitation).

## 4 Conclusion

These results show the utility of a methanol-based SEC system with viscometric detection for the analysis of PNIPAM linear and branched polymers.

## Additional information

The authors declare no competing financial interests.

## Funding

This research was undertaken in part using funding granted by the Wellcome Trust medical charity (post-doctoral fellowship for Swift, 0998800/B/12/Z); EPSRC (PhD studentship for Plenderleith) Innovate UK/Smith and Nephew Ltd. (TSB 101224) and MRC (MR/N501888/2) (post doctoral fellowships for Hoskins).

## Appendix A Supplementary data

Electronic Supporting information provided can be found including full hydrodynamic radii of polymers disclosed in Table 1, details of calibration of THF SEC system, further characterization and analytical data of PNIPAM standards, detail of DOSY NMR technique, synthesis of iron ATRP polymerization, performance of PEO standards on methanol SEC, performance of polargel columns with THF as eluent, alpha values of HB-PNIPAM polymers, full moments of size distribution of HB-PNIPAM samples using both SEC and ^1^H DOSY. Raw data will be uploaded to Bradford Scholars repository for online access.
