# Supplementary material for: Analysis using size exclusion chromatography of poly(N-isopropyl acrylamide) using methanol as an eluent
Source: J Chromatogr A. 2017 Jul 28;1508:16–23. doi: 10.1016/j.chroma.2017.05.050 (PMC5486375; doi:10.1016/j.chroma.2017.05.050)
Supplement: Supplementary file 1 [file mmc1.docx]

Methanol Size Exclusion Chromatography: First Example of an Alcohol Based Size Exclusion Chromatography Capable of Resolving Multi-Functional Highly-Branched Polymers.

T. Swift^a^, R. Hoskins^a^, D. Pownall^a^, R. Telford^b^, R. Plenderleith^b^ and S. Rimmer^a*^

**Additional Supporting Information**

# Contents of the Supporting Information:

1. Hydrodynamic radii of PNIPAM Polymers in SEC Solvents
2. Calibration of SEC with Tetrahydrofuran (THF) as eluent with crosslinked polystyrene stationary phase.
3. Characterisation of PNIPAM standards
4. Analysis of PNIPAM standards
5. Using Diffusion NMR to give accurate intrinsic viscosities
6. Calibration of Agilent PolarGel Columns Using PNIPAM Stds.
7. Preparation of Linear PNIPAM using Iron based ATRP
8. Performance of poly(ethylene oxide) Standards using SEC with methanol as eluent
9. Examination of the use of PolarGel Stationary Phases for SEC with THF as eluent
10. Fitting alpha values across full HB-PNIPAM Molar Mass Distribution
11. 1H DOSY and SEC of HB-PNIPAM in MeOD
12. Column Retention Time (Toluene Injection)
13. High Molar Mass Excluded Samples
14. System Calibration Reports
15. Supporting Information References

# 1. Hydrodynamic Radii of PNIPAM Polymers in SEC Solvents

The hydrodynamic radii of linear PNIPAM polymers **1 – 8** were determined in triplicate, using DOSY NMR, and the average values are shown in Table 1 in the main manuscript. The raw data for this table are shown below in Table S1, S2 and Fig. S1.

Table S1 – Hydrodynamic radii of polymers in deuterated solvents, averages and Std. Dev

| **M_n_** | **Averages** | | | **Std. Dev** | | |
| --- | --- | --- | --- | --- | --- | --- |
|  | D_2_O | MeOD | THF | D_2_O | MeOD | THF |
| 1405500 | 13.52 | 13.32 | 13.12 | 0.01 | 0.11 | 0.05 |
| 240050 | 8.07 | 7.32 | 7.78 | 0.02 | 0.02 | 0.10 |
| 238500 | 6.76 | 6.50 | 6.56 | 0.01 | 0.01 | 0.05 |
| 122280 | 5.51 | 4.87 | 5.06 | 0.00 | 0.06 | 0.05 |
| 45000 | 3.56 | 3.36 | 3.25 | 0.02 | 0.08 | 0.09 |
| 39000 | 3.39 | 3.02 | 3.01 | 0.01 | 0.05 | 0.03 |
| 28000 | 3.16 | 2.63 | 2.73 | 0.02 | 0.04 | 0.05 |
| 18500 | 2.57 | 2.29 | 2.31 | 0.01 | 0.01 | 0.02 |
| 14000 | 2.24 | 2.09 | 2.11 | 0.01 | 0.03 | 0.04 |

RM two way paired ANOVA in Graphpad Prism of the data indicated a statistically significant P value (< 0.001) variation source for both the molar mass (99.67% of total variation) and Solvent (0.2324% of total variation):

| Two-way RM ANOVA | Matching: Stacked |  |  |  |  |
| --- | --- | --- | --- | --- | --- |
| Alpha | 0.05 |  |  |  |  |
|  |  |  |  |  |  |
| Source of Variation | % of total variation | P value | P value summary | Significant? |  |
| Interaction | 0.07776 | <0.0001 | **** | Yes |  |
| Molar Mass | 99.67 | <0.0001 | **** | Yes |  |
| Solvent | 0.2324 | <0.0001 | **** | Yes |  |
| Subjects (matching) | 0.001652 | 0.4641 | ns | No |  |
|  |  |  |  |  |  |
| ANOVA table | SS | DF | MS | F (DFn, DFd) | P value |
| Interaction | 1.224 | 16 | 0.07648 | F (16, 96) = 34.94 | P<0.0001 |
| Molar Mass | 1569 | 8 | 196.1 | F (8, 96) = 89585 | P<0.0001 |
| Solvent | 3.657 | 2 | 1.828 | F (2, 12) = 843.7 | P<0.0001 |
| Subjects (matching) | 0.026 | 12 | 0.002167 | F (12, 96) = 0.9901 | P=0.4641 |
| Residual | 0.2101 | 96 | 0.002189 |  |  |
|  |  |  |  |  |  |
| Number of missing values | 0 |  |  |  |  |

Table S2 – Raw data from 5 repeats of Hydrodynamic Radii of polymers in deuterated solvents

| **M_n_** | **D_2_O** | | |  | | |
| --- | --- | --- | --- | --- | --- | --- |
|  | 1 | 2 | 3 | 4 | 5 |  |
| 1405500 | 13.51 | 13.515 | 13.53 | 13.518 | 13.536 |  |
| 240050 | 8.071 | 8.052 | 8.108 | 8.079 | 8.058 |  |
| 238500 | 6.746 | 6.764 | 6.768 | 6.7652 | 6.7602 |  |
| 122280 | 5.507 | 5.507 | 5.506 | 5.509 | 5.51 |  |
| 45000 | 3.556 | 3.597 | 3.543 | 3.561 | 3.555 |  |
| 39000 | 3.388 | 3.388 | 3.388 | 3.386 | 3.399 |  |
| 28000 | 3.154 | 3.133 | 3.184 | 3.16 | 3.154 |  |
| 18500 | 2.57 | 2.576 | 2.552 | 2.571 | 2.571 |  |
| 14000 | 2.243 | 2.243 | 2.233 | 2.244 | 2.256 |  |
| **M_n_** | **MeOD** | | |  | | |
|  | 1 | 2 | 3 | 4 | 5 |  |
| 1405500 | 13.13 | 13.35 | 13.362 | 13.378 | 13.391 |  |
| 240050 | 7.318 | 7.302 | 7.352 | 7.336 | 7.308 |  |
| 238500 | 6.502 | 6.5 | 6.499 | 6.487 | 6.506 |  |
| 122280 | 4.835 | 4.802 | 4.88 | 4.92 | 4.933 |  |
| 45000 | 3.431 | 3.231 | 3.431 | 3.331 | 3.386 |  |
| 39000 | 3.044 | 3.093 | 2.947 | 2.997 | 3.001 |  |
| 28000 | 2.625 | 2.668 | 2.585 | 2.601 | 2.686 |  |
| 18500 | 2.293 | 2.283 | 2.283 | 2.291 | 2.299 |  |
| 14000 | 2.063 | 2.077 | 2.077 | 2.101 | 2.141 |  |
| **M_n_** | **THF** | | |  | | |
|  | 1 | 2 | 3 | 4 | 5 |  |
| 1405500 | 13.115 | 13.06 | 13.075 | 13.163 | 13.173 |  |
| 240050 | 7.841 | 7.831 | 7.84 | 7.621 | 7.744 |  |
| 238500 | 6.554 | 6.606 | 6.611 | 6.561 | 6.48 |  |
| 122280 | 5.039 | 5.133 | 5.092 | 5.034 | 5.022 |  |
| 45000 | 3.121 | 3.322 | 3.239 | 3.228 | 3.336 |  |
| 39000 | 2.995 | 2.995 | 3.057 | 3.026 | 2.998 |  |
| 28000 | 2.808 | 2.706 | 2.751 | 2.699 | 2.705 |  |
| 18500 | 2.314 | 2.349 | 2.295 | 2.298 | 2.301 |  |
| 14000 | 2.159 | 2.13 | 2.14 | 2.066 | 2.077 |  |

Fig S1 – Hydrodynamic Radii of linear PNIPAM polymers in deuterated solvents.

Paired T-Tests of this data show that the deuterated D_2_O data are significantly different (P > 0.001) from the other two datasets, whilst the methanol and THF data are not significantly different (P = 0.3713), as shown below:

| Table Analyzed | Data 1 |
| --- | --- |
|  |  |
| Column C | THF |
| vs. | vs. |
| Column B | MeOD |
|  |  |
| Paired t test |  |
| P value | 0.3713 |
| P value summary | ns |
| Significantly different (P < 0.05)? | No |
| One- or two-tailed P value? | Two-tailed |
| t, df | t=0.9471 df=8 |
| Number of pairs | 9 |
|  |  |
| How big is the difference? |  |
| Mean of differences | 0.05898 |
| SD of differences | 0.1868 |
| SEM of differences | 0.06227 |
| 95% confidence interval | -0.08463 to 0.2026 |
| R squared (partial eta squared) | 0.1008 |
|  |  |
| How effective was the pairing? |  |
| Correlation coefficient (r) | 0.9987 |
| P value (one tailed) | <0.0001 |
| P value summary | **** |
| Was the pairing significantly effective? | Yes |

# 2. Calibration of SEC with Tetrahydrofuran (THF) as eluent with crosslinked polystyrene stationary phase.

SEC was conducted using PLGel (mixed B) stationary phase (3 x 300 cm columns) calibrated with Polystyrene standards (Agilent EasiVial) using an RI and viscometric detectors (Agilent 1260). The eluent was THF containing 1 mol % tetrabutyl ammonium bromide (TBAB 1 wt%). This was used to determine the accurate molar mass distributions of linear PNIPAM standard polymers.

Fig. S2 – Calibration of THF SEC with Agilent EasiVal Poly(styrene) standards

Table S3 – Calibration of THF GPC with Agilent EasiVial poly(styrene) standards

| RT Corrected (mins) | Log M | [η] (dL/g) | Log (M * [η]) | Calibration [Log (M *[η])] |
| --- | --- | --- | --- | --- |
| 17.38333 | 6.761552 | 8.419 | 7.686812 | 7.67051 |
| 18.1 | 6.478711 | 4.7639 | 7.156673 | 7.177088 |
| 19.33333 | 5.996512 | 2.1965 | 6.338243 | 6.360477 |
| 20.31667 | 5.65925 | 1.2216 | 5.746179 | 5.733794 |
| 21.21667 | 5.342817 | 0.7552 | 5.220879 | 5.174984 |
| 22.45 | 4.870696 | 0.346 | 4.409773 | 4.425584 |
| 24 | 4.310906 | 0.136 | 3.444445 | 3.49794 |
| 24.9 | 4.030195 | 0.09 | 2.984437 | 2.960066 |
| 25.66667 | 3.744293 | 0.0593 | 2.517348 | 2.499048 |
| 27.05 | 3.170262 | 0.0328 | 1.686136 | 1.652594 |
| 27.76667 | 2.835691 | 0.0231 | 1.199303 | 1.204345 |

**3. Characterisation of PNIPAM standards**

Following synthesis of linear PNIPAM standards they were characterized by NMR and FTIR as outlined in the manuscript. Further characterization details are provided below. PNIPAM 1 – 7 gave identical responses in all respects save molar mass distributions and diffusion measurements as they are chemically identical.


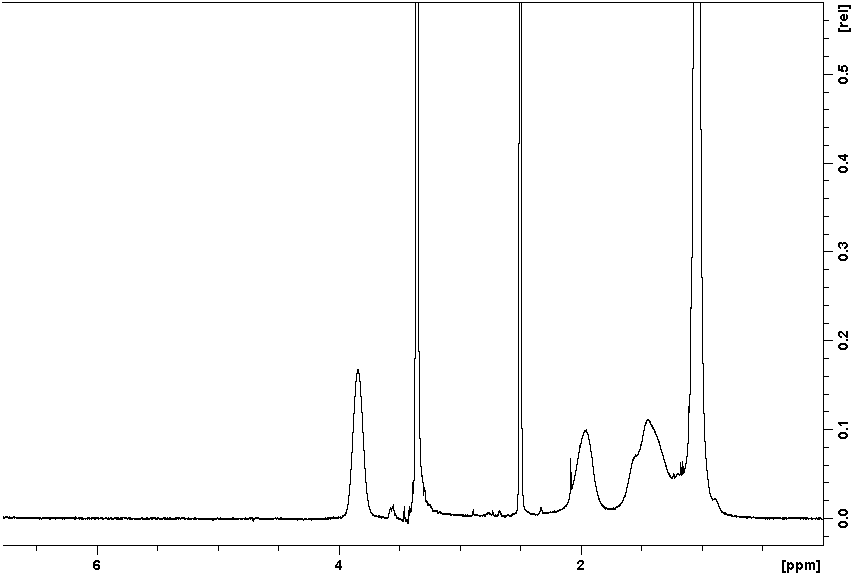


Fig 1A – ^1^H NMR of PNIPAM 7 IN D_2_O – screenshot from Bruker Topspin


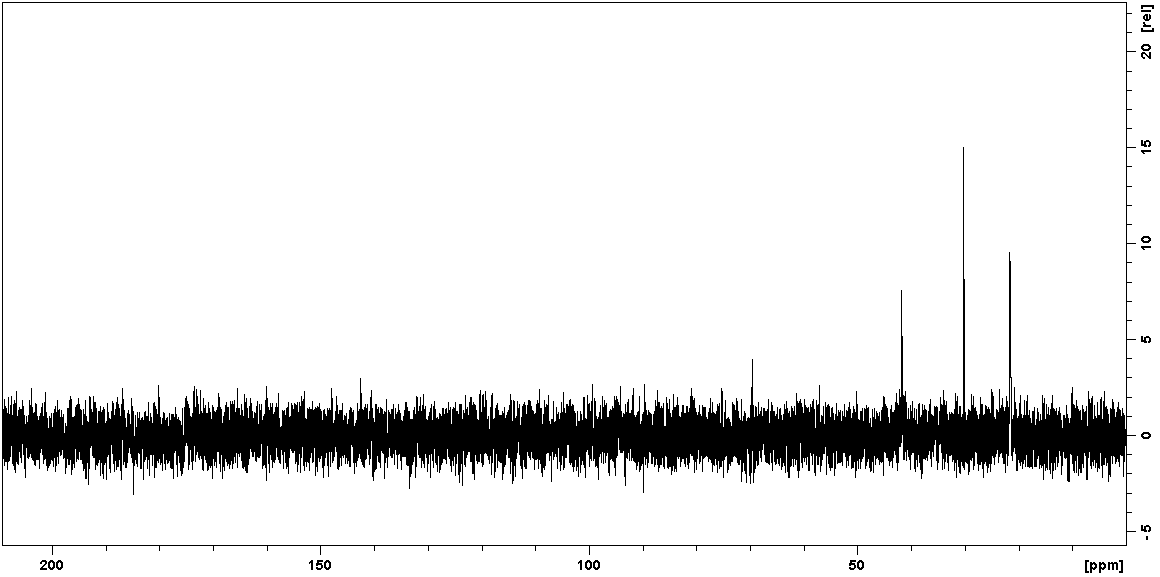


Fig 1A2 – ^13^C NMR of PNIPAM 7 in D_2_O – screenshot from Bruker Topspin


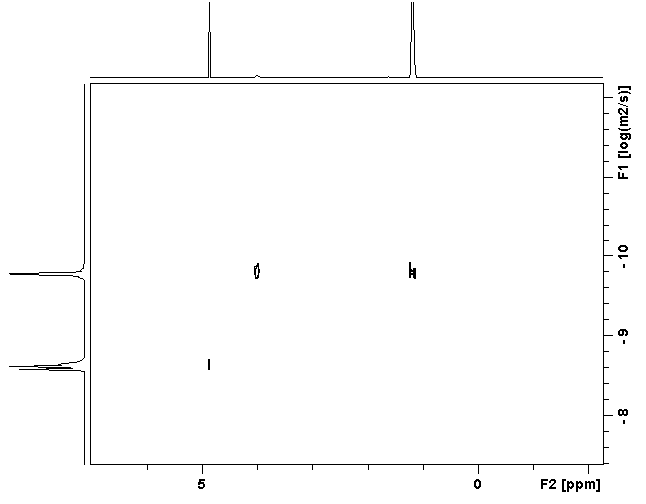


Fig 1B – Zoomed Out ^1^H DOSY NMR of PNIPAM 7 in D_2_O
– screenshot taken from Bruker Topspin

Fig 1B2 – Extracted diffusion of PNIPAM 7 in D_2_O polymer and solvent.


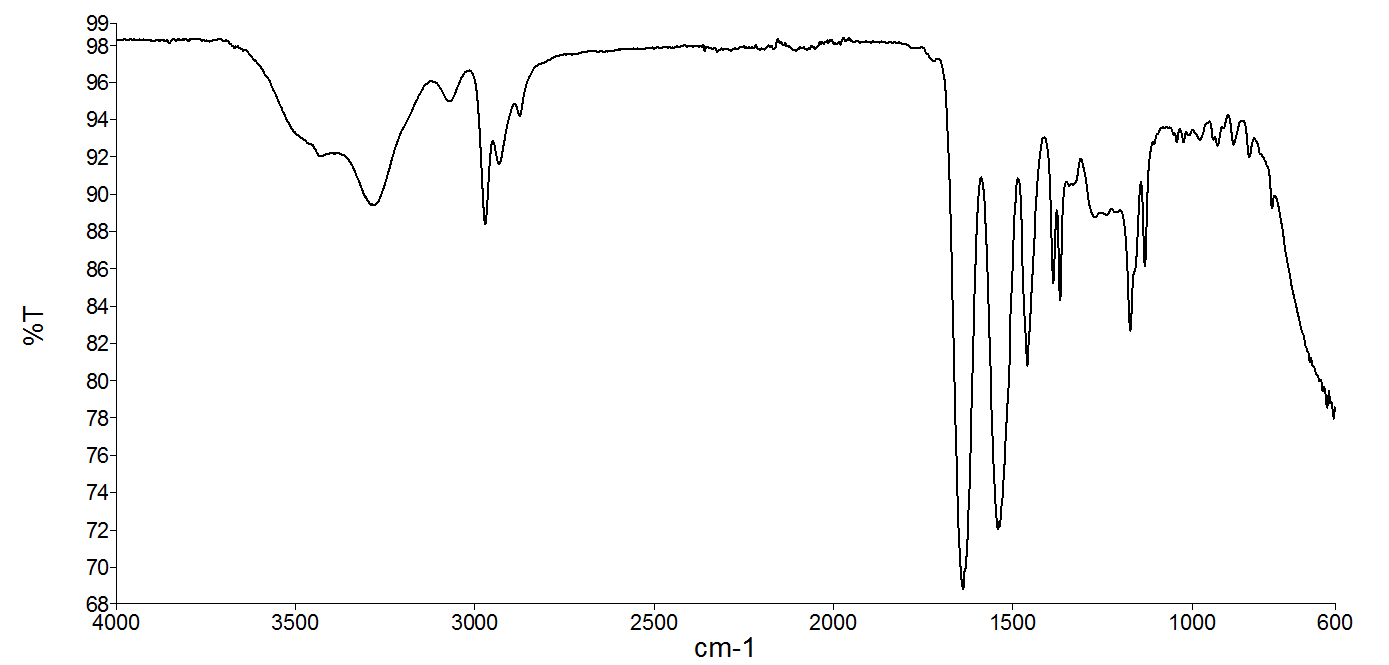


Fig 1C – FTIR of PNIPAM 7 (solid phase)

Peak identification in cm^-1^: 3300 broad OH + NH, 2980 CH, 1646 Amide I, 1550 Amide II, 1460 CH_2_, 1387 Isopropyl, 1367 Isopropyl, 1170 C-H.

Data used in this study will be uploaded to Bradford Scholars (https://bradscholars.brad.ac.uk) repository for free, universal access in perpetuity.

# 4. Analysis of PNIPAM standards

Following calibration of the THF (+TBAB) SEC using PLGel stationary phase, it was used to measure the molar mass of linear PNIPAMs 1 - 7. The molar mass distributions (Fig. S3) were calculated using the Universal calibration with polystyrene standards.

Fig S3 – Molar mass distributions of linear PNIPAM polymers (dw/dlog M – left axis)
with retention time (minutes - right axis, red markers) in THF

Full characterisation data is shown below in Table S4.

Table S4 – Expansion of Table 1 data of PNIPAM standards

| Sample | M : I^a^ | M_p_ | M_n_ | M_w_ | M_z_ | Ð^b^ |
| --- | --- | --- | --- | --- | --- | --- |
| PNIPAM 1 | 9,600: 1 | 1,405.50 | 2,943.52 | 6,442.16 | 11,839.17 | 2.18 |
| PNIPAM 2 | 800 : 1 | 240.05 | 128.63 | 294.10 | 521.59 | 2.29 |
| PNIPAM 3 | 600 : 1 | 238.50 | 238.37 | 336.53 | 409.71 | 1.41 |
| PNIPAM 4 | 400 : 1 | 122.28 | 55.08 | 116.77 | 241.99 | 2.12 |
| PNIPAM 5 | 200 : 1 | 45.00 | 41.06 | 82.62 | 156.04 | 2.01 |
| PNIPAM 6 | 100 : 1 | 39.00 | 34.81 | 77.91 | 160.49 | 2.24 |
| PNIPAM 7 | 100 : 1 | 28.00 | 22.61 | 53.01 | 109.54 | 2.34 |
| PNIPAM 8 | 100 : 1 | 18.50 | 18.73 | 38.87 | 83.38 | 2.08 |
| PNIPAM 9 | 50 : 1 | 14.00 | 15.98 | 28.95 | 53.83 | 1.81 |

# 5. Using Diffusion NMR to give accurate intrinsic viscosities

Intrinsic viscosity can be determined from diffusion NMR measurements using the Stokes-Einstein relationship. This was validated using poly(ethylene glycol) standards (Agilent Technologies), the properties of which are shown in Table S5. According to the specification supplied by Agilent, the molar masses were determined by SEC with a refractive index detector on PL aquagel-OH mixed-H 8m columns at ambient temperature. An aqeuous mobile phase with 0.02 NaN_3_ salt at 1 ml min^-1^ was used. 100 μl volumes were injected at 0.025% concentration to determine M_P_, M_N_, M_w_, M_V_ and Ð. This was then confirmed using a light scattering detector (390-MDS 15/90 LS) to give independent confirmation of M_W_

Table S5 – Properties of Poly(ethylene glycol) / poly(ethylene oxide) standards used to test intrinsic viscosity measurements..

| **M_P_*** | **M_N_*** | **M_W_*** | **M_V_*** | **Ð*** | **M_W_ (LS)^#^** |
| --- | --- | --- | --- | --- | --- |
| 610.00 | 580 | 620 | 610 | 1.07 | 630 |
| 3870.00 | 3730 | 3830 | 3820 | 1.03 | 3580 |
| 16100.00 | 14330 | 15260 | 15130 | 1.06 | 15870 |
| 73550.00 | 68850 | 71650 | 71250 | 1.04 | 80400 |
| 552000.00 | 493500 | 550500 | 542500 | 1.12 | 622,000 |
| 863500.00 | 668500 | 774000 | 762500 | 1.16 | 875000 |
| 1522000.00 | 1179000 | 1358000 | 1336000 | 1.15 | 1243000 |

Diffusion measurements were carried out using a supplied ledbpbgp2s_compensated program (Bruker), modified to run over 32 gradient steps. The pulse, delay sequence is shown below in Fig S5.


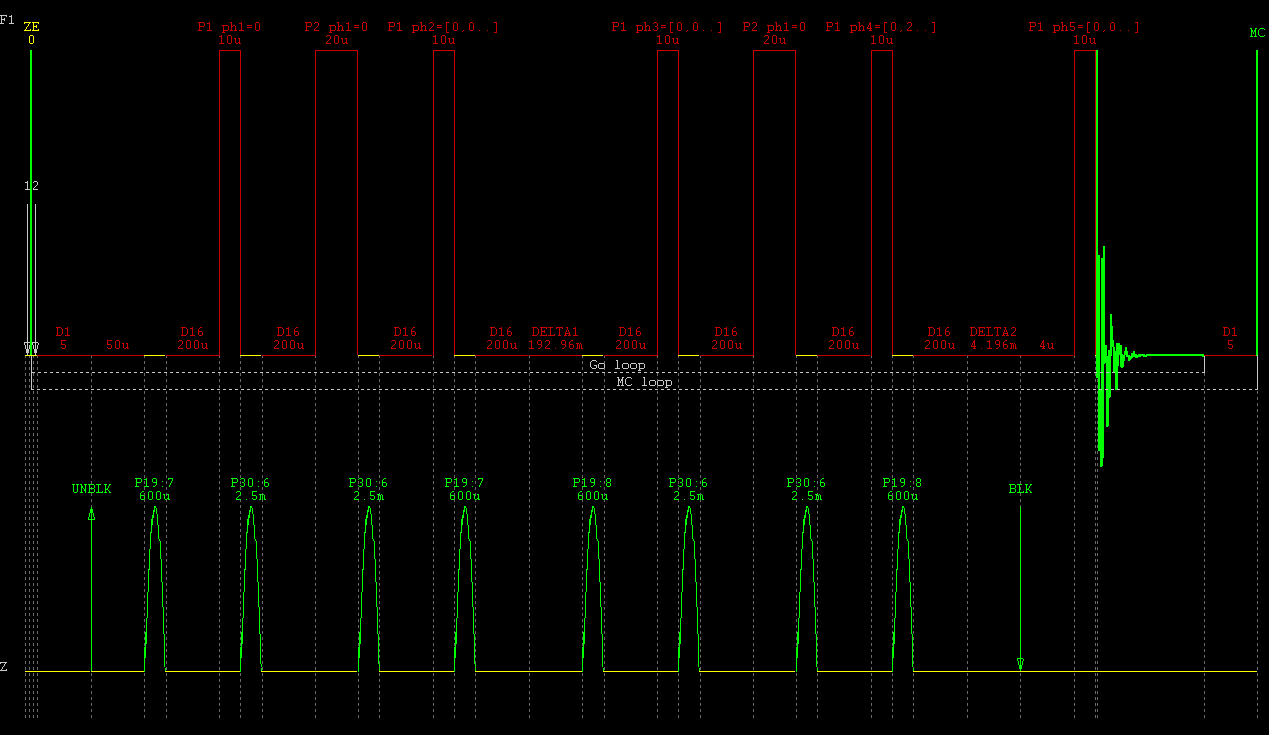


Fig. S4 – Bruker Pulse Program

Additionally it is worth considering that DOSY can be affected by convection internally within the NMR tubes[^1^](#_ENREF_1). Studies have shown that this is less problematic for samples in deuterium oxide than for organic solvents such as chloroform or methanol[^1^](#_ENREF_1). As such all measurements carried out in deuterated solvents used thin NMR tubes (0.2 mm diameter) to reduce this issue. Temperature accuracy was ensured by initial calibration of the sample temperature by measurement of the shift between the residual OH and CH_3_ resonances of methanol (99.8% MeOD).

Calibration of the gradient field strength was performed using a sample of H_2_O in D_2_O (1% v/v) doped with GdCl_3_ (0.1 mg) as a paramagnetic relaxation agent. Gradient strengths were calibrated to provide a diffusion coefficient of 1.91 x 10^-9^ m^-2^ s^-1^ at 298.15 K (gradients operated from 95% to 5% using 16 points with a quadratic decay). The same bipolar LED sequence as for the sample measurements was used, with sine shaped gradient pulses and gradient strengths incremented between 0.28 and 5.19 G mm^-1^ in 16 steps equally spaced in gradient squared.

Samples were prepared in D_2_O at 1 mg ml^-1^, with brief heating to 60 °C to encourage complete dissolution. Tubes were filled with 0.8 ml solution to a constant volume. One dimensional 1H experiments were recorded using 16 scans. ^1^H diffusion measurements were recorded using an LED sequence with bipolar gradients, with sine shaped gradient strength incremented between 0.28 and 5.19 G mm^-1^ in 32 steps equally spaced linearly. Data analyses were performed using TopSpin software version 3.5 (patch level 5).

As the signal decay time, and therefore the number of data points, varies between sample constituents; it is not possible to fully optimize the system with respect to viscosity and diffusion of solvated polymers with one measurement. For this study some pre-optimized settings were employed which adequately characterize many samples, giving fast and efficient gradient decays in a short time period. In order to study as wide a variety of diffusions as possible a linear gradient ramp was employed, as opposed to a quadratic ramp which is more useful for studying small ranges of known samples.

The diffusion of poly(ethylene oxide) gives an extremely simple NMR spectrum with only 1 proton NMR peak arising from the hydrophobic polymer backbone (3.63 ppm) at a distinct position from residual protons in the solvent resonance (4.70 ppm). This peak is shown along the x axis of a diffusion spectrum, displayed at the top of Fig (S6). The Y axis of the raw dataset indicates the log of the diffusion, with both the solvent and the polymer demonstrating distinct diffusion distributions. A contour map of these 2 distributions (solvent and polymer) is overlaid and separation between the molecules of distinct sizes becomes readily apparent.


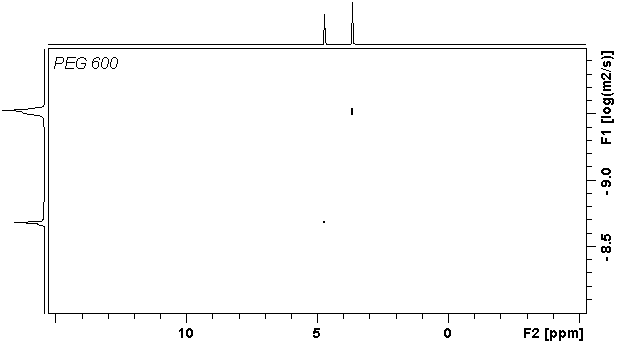


Fig. S5 – Raw diffusion spectrum of poly(ethylene oxide) (M_n_ 580 mol-1) in D2O, with polymer backbone peak (3.63 ppm, -9.55 logD) distinct from solvent diffusion (4.70 ppm, -8.69 logD)

In order to ensure the accuracy of using the Stokes Einstein equation to get the hydrodynamic radius of the polymer within the NMR tube a correction factor was used regarding the solvent diffusion peak. A blank sample of D_2_O was scanned and gave a diffusion value of 1.914 x 10^-9^ m^2^ S^-1^. This measurement was repeated several times (n = 3) to ensure accuracy and found to vary by less than 1%. The value is close to the previously measured values for D_2_O / H_2_O mixtures.[^2-3^](#_ENREF_2) The number was used to gauge both the diffusion and viscosity of the pure solvent, and then when the diffusion of the solvent with polymer was analysed the viscosity of the sample was determined thus:

$$D_{1}ɳ_{1}= D_{2}ɳ_{2}$$

This correction ensured that all hydrodynamic radii calculated using Stokes-Einstein would be accurate. The diffusion values of these polymers were recorded and the hydyodynamic radius calculated (Table S6). These hydrodynamic radii were then used to calculate the intrinsic viscosity of the polymers in aqueous solution at 25 °C. Literature data were then gathered and no significant difference was observed between the intrinsic viscosity of the PEO/PEG standards using the DOSY method to published literature values [^4-10^](#_ENREF_4).

Table S6 – Diffusion values for polymer standards and solvent used to calculate sample viscosity and hydrodynamic radius.

| Sample | M_n_  g mol^-1^ | D_H2O_ m^2^ S^-1^ x 10^-9^ | D_P_ m^2^ S^-1^ x 10^-9^ | $ɳ$  kg m^-1^ S^-1^ | R_H_ nm |
| --- | --- | --- | --- | --- | --- |
| 1 | 580 | 2.0559 | 0.2818 | 0.0010 | 0.75 |
| 2 | 3730 | 2.2594 | 0.1069 | 0.0009 | 2.17 |
| 3 | 14330 | 2.2387 | 0.0550 | 0.0010 | 4.18 |
| 4 | 68850 | 1.8197 | 0.0174 | 0.0012 | 10.74 |
| 5 | 493500 | 2.0417 | 0.0069 | 0.0010 | 30.26 |
| 6 | 668500 | 2.2131 | 0.0063 | 0.0010 | 35.97 |
| 7 | 1179000 | 2.5704 | 0.0062 | 0.0008 | 52.17 |

R_H_ values were plotted and compared to known values in the literature (Fig. S7). Comparisons in the datasets were given by compiling all available literature data into one logarithmic fit (N = 54, slope 0.5448 ± 0.005958, Y Intercept -1.659 ± 0.02621, R2 0.9938) and the 7 datapoints gathered from the DOSY measurements into another (N = 7, slope 0.5450 ± 0.006271, Y Intercept - -1.638 ± 0.03063, R2 9993). Comparison of these slopes shows that the 2 fits were identical in both slope (P = 0.9722) and intercept (P = 0.2893). The regression analysis shows that there was no statistical difference between the datasets.

Literature data used in Fig. 2, in the main article, for the intrinsic viscosities were derived from several literature sources. Most of these sources quoted Mw but two sources provided M_N_ and M_v_ data, however these are assumed to be narrow molecular weight distribution polymers as the results appear similar and so are still included for reference. Inferred hydrodynamic radii of polymers from publications that only quote R_H_ values are not used. Extracted data from these publications are shown below:

Fig. 2 [η] of PEO / PEG of varying molar masses from previous studies[^4-10^](#_ENREF_4)

|  | M | Ð | [η] |  |  | Mw | Ð | [η] |
| --- | --- | --- | --- | --- | --- | --- | --- | --- |
|  | / g mol^-1^ | (If provided) | dl g^-1^ |  |  | / g mol^-1^ | (If provided) | dl g^-1^ |
| Armstrong[^7^](#_ENREF_7) | 1450 | Near MD | 0.0629 |  | Amu[^4^](#_ENREF_4) | 4500 | Fraction | 0.11 |
| M_W_ | 2000 | Near MD | 0.0788 |  | M_N_ | 7746 | Fraction | 0.195 |
|  | 3350 | Near MD | 0.107 |  |  | 13470 | Fraction | 0.283 |
|  | 5000 | Near MD | 0.1517 |  |  | 22445 | Fraction | 0.489 |
|  | 6450 | Near MD | 0.1486 |  |  | 37640 | Fraction | 0.759 |
|  | 7500 | Near MD | 0.1937 |  |  |  |  |  |
|  | 8500 | Near MD | 0.1837 |  |  |  |  |  |
|  | 11840 | Near MD | 0.2259 |  | Kirincic^9^ | 300 | 1.35* | 0.0392 |
|  | 22800 | Near MD | 0.3181 |  | M_n_ | 400 | 1.46* | 0.0451 |
|  | 29600 | Near MD | 0.4545 |  |  | 600 | 1.25* | 0.0502 |
|  | 35000 | Near MD | 0.5128 |  |  | 900 | 1.14* | 0.0578 |
|  | 100000 | Near MD | 1.073 |  |  | 1000 | 1.34* | 0.0661 |
|  | 300000 | Near MD | 2.668 |  |  | 1500 | 1.18* | 0.0763 |
|  | 600000 | Near MD | 3.745 |  |  | 2000 | 1.18* | 0.0896 |
|  | 1000000 | Near MD | 4.528 |  |  | 3000 | 1.17* | 0.1130 |
|  |  |  |  |  |  | 4000 | 1.24* | 0.1398 |
| Kawaguchi 1[^5^](#_ENREF_5) | 424 | 1.06 | 0.040 |  |  | 6000 | 1.14* | 0.1714 |
| M_W_ | 825 | 1.10 | 0.055 |  |  | 10000 | 1.21* | 0.2492 |
|  | 2075 | 1.07 | 0.087 |  |  | 12000 | 1.60* | 0.3414 |
|  | 3990 | 1.05 | 0.130 |  |  | 15000 | 1.40* | 0.3626 |
|  | 5559 | 1.09 | 0.152 |  |  | 20000 | 1.29* | 0.4195 |
|  | 2240 | 1.15 | 0.395 |  |  | 35000 | 1.10* | 0.5529 |
|  |  |  |  |  |  |  |  |  |
| Kawaguchi 2[^6^](#_ENREF_6) | 40 | 1.03 | 0.0058 |  | Fabula[^10^](#_ENREF_10) | 200000 | - | 1.7 |
| M_W_ | 70 | 1.02 | 0.00894 |  | M_V_ | 450000 | - | 3.2 |
|  | 150 | 1.04 | 0.0144 |  |  | 3900000 | - | 16.0 |
|  | 660 | 1.10 | 0.04 |  |  | 5800000 | - | 24.0 |
|  | 1200 | 1.12 | 0.0632 |  |  | 8000000 | - | 31.0 |
|  |  |  |  |  |  |  |  |  |
| Woodley[^8^](#_ENREF_8) | 86000 | MD | 0.86 |  |  |  |  |  |
| M_W_ | 160000 | MD | 1.41 |  |  |  |  |  |
|  | 252000 | MD | 2.27 |  |  |  |  |  |
|  | 594000 | MD | 3.97 |  |  |  |  |  |
|  | 838000 | MD | 5.29 |  |  |  |  |  |
|  | 860000 | MD | 5.40 |  |  |  |  |  |
|  | 996000 | MD | 6.33 |  |  |  |  |  |

* several papers that do not site dispersity and merely claim samples are ‘monodisperse’.

Not all samples provide sufficient information to make an informed judgement about sample size distribution. The Armstrong paper describes the POE / PEG samples as ‘low dispersity’ compared to other polymers in the paper and additionally calls them ‘near monodisperse’ but provides no data to evidence this. The data provided by Woodley is described as ‘monodisperse’ but no dispersity data is presented. Amu data is described as fractions of PEO. Kirincic data (*) does not provide Mw / Mn but does provide M_V_ / M_N_ so the quoted dispersity is not comparable to Kawaguchi data.

# 6. Calibration of Agilent PolarGel Columns Using PNIPAM Stds.

Raw data used in determination of Mn, Mw, Ð and [ɳ] for Methanol GPC Calibration


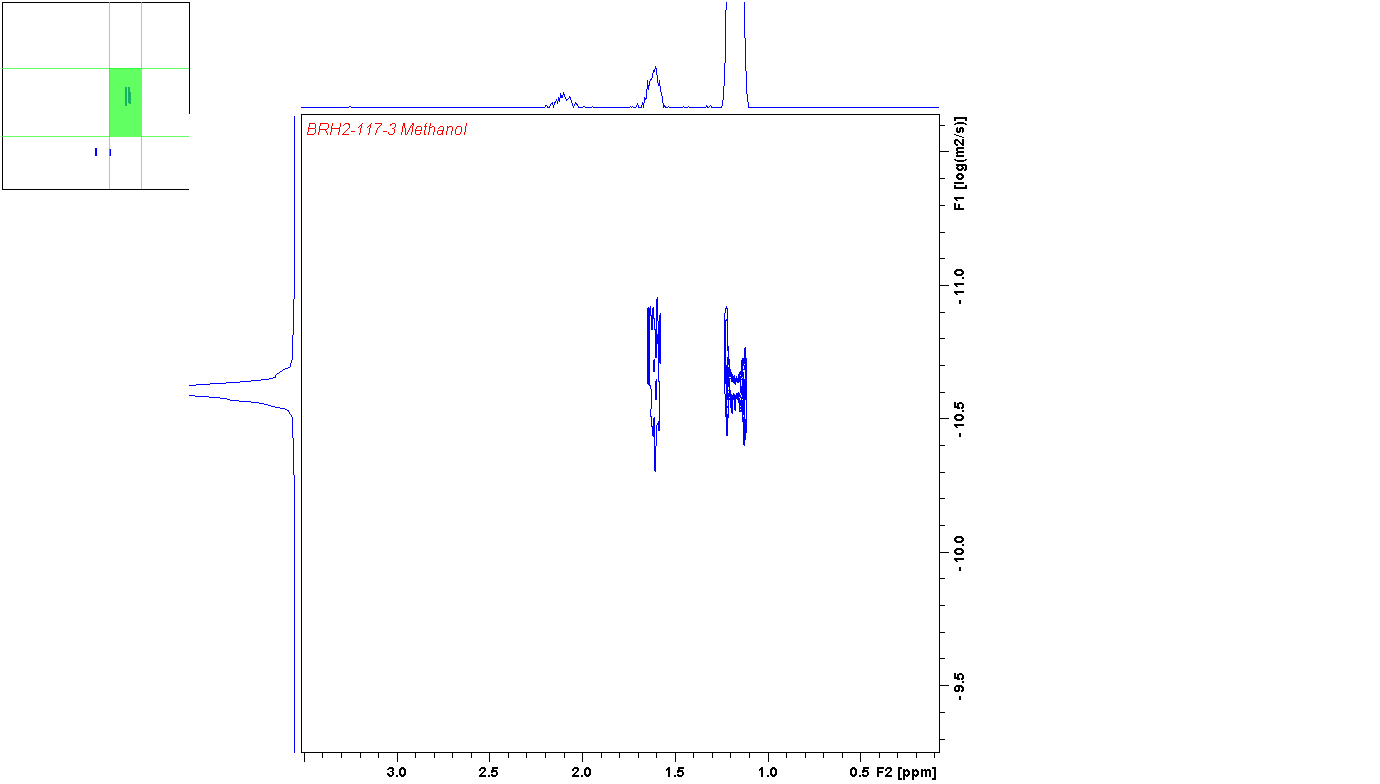


Fig. S6 – Raw chromatograms of PNIPAM 1 to 9 in Methanol

Table S8 – Raw DOSY Data of Polymer diffusion on Methanol

| Polymer 1 DOSY NMR | 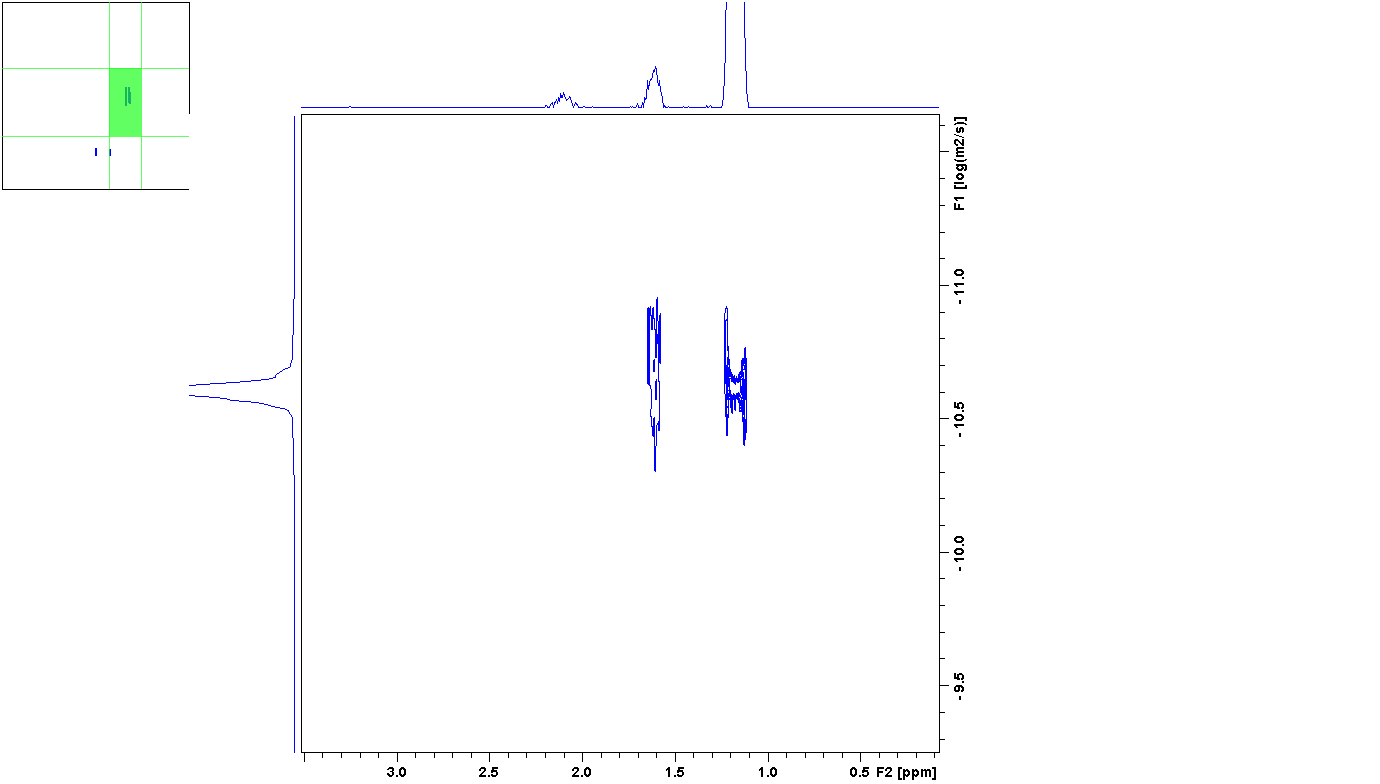 |
| --- | --- |
| Polymer 2 DOSY NMR | 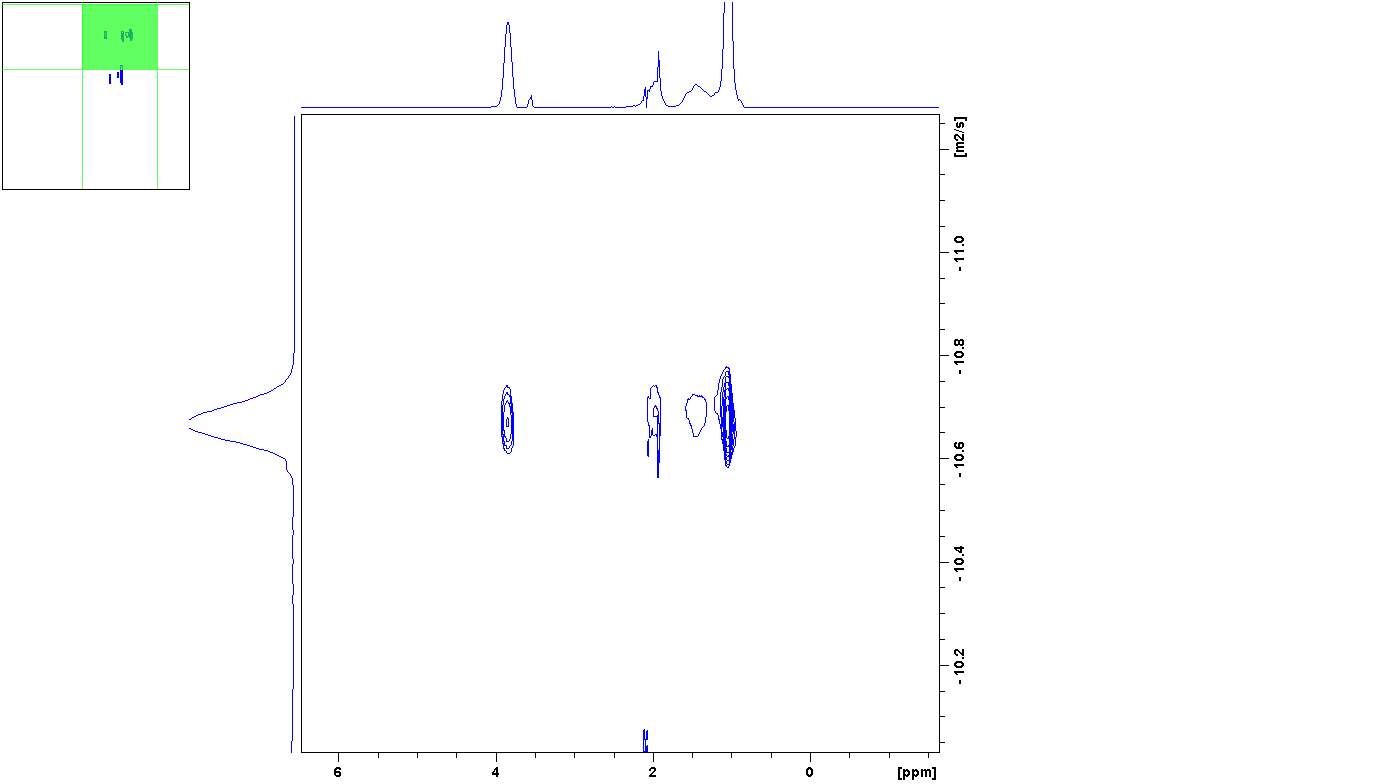 |
| Polymer 3 DOSY NMR | 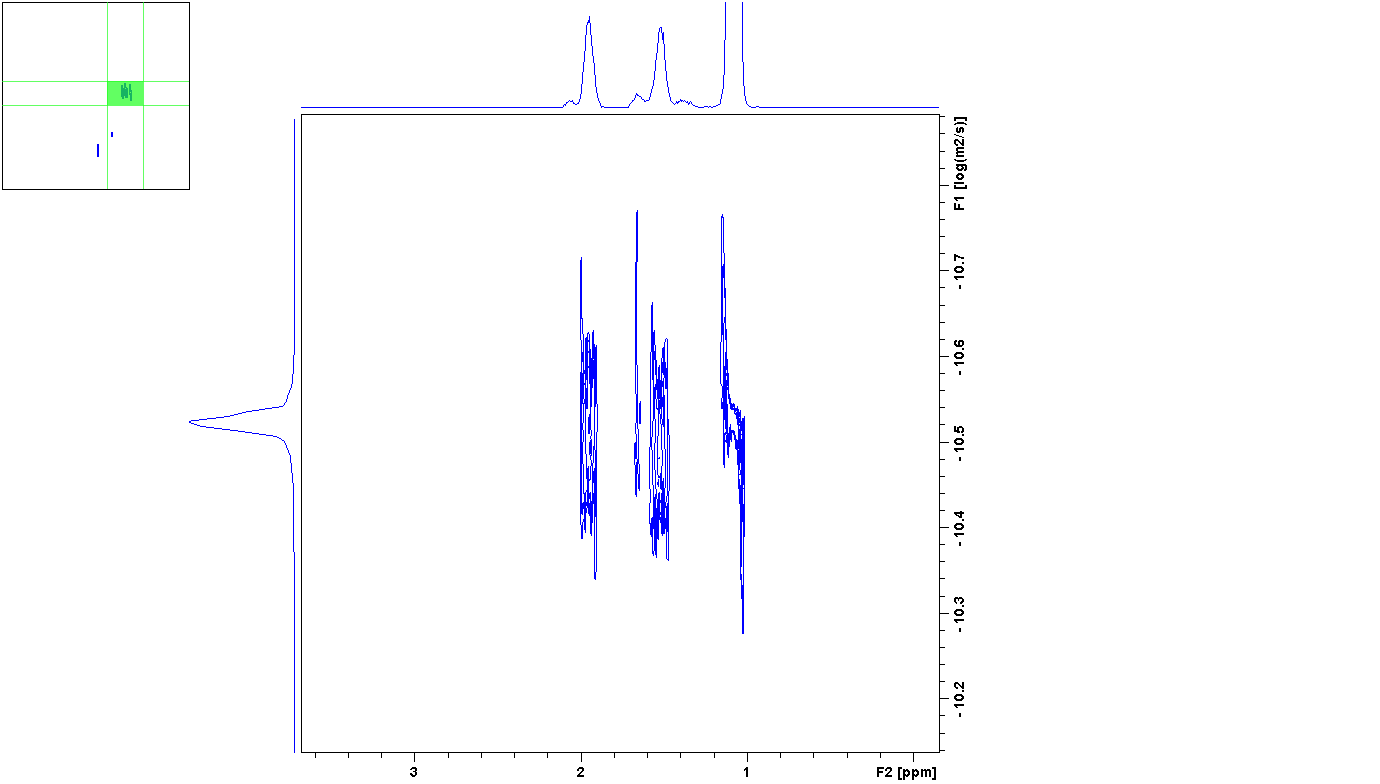 |
| Polymer 4 DOSY NMR | 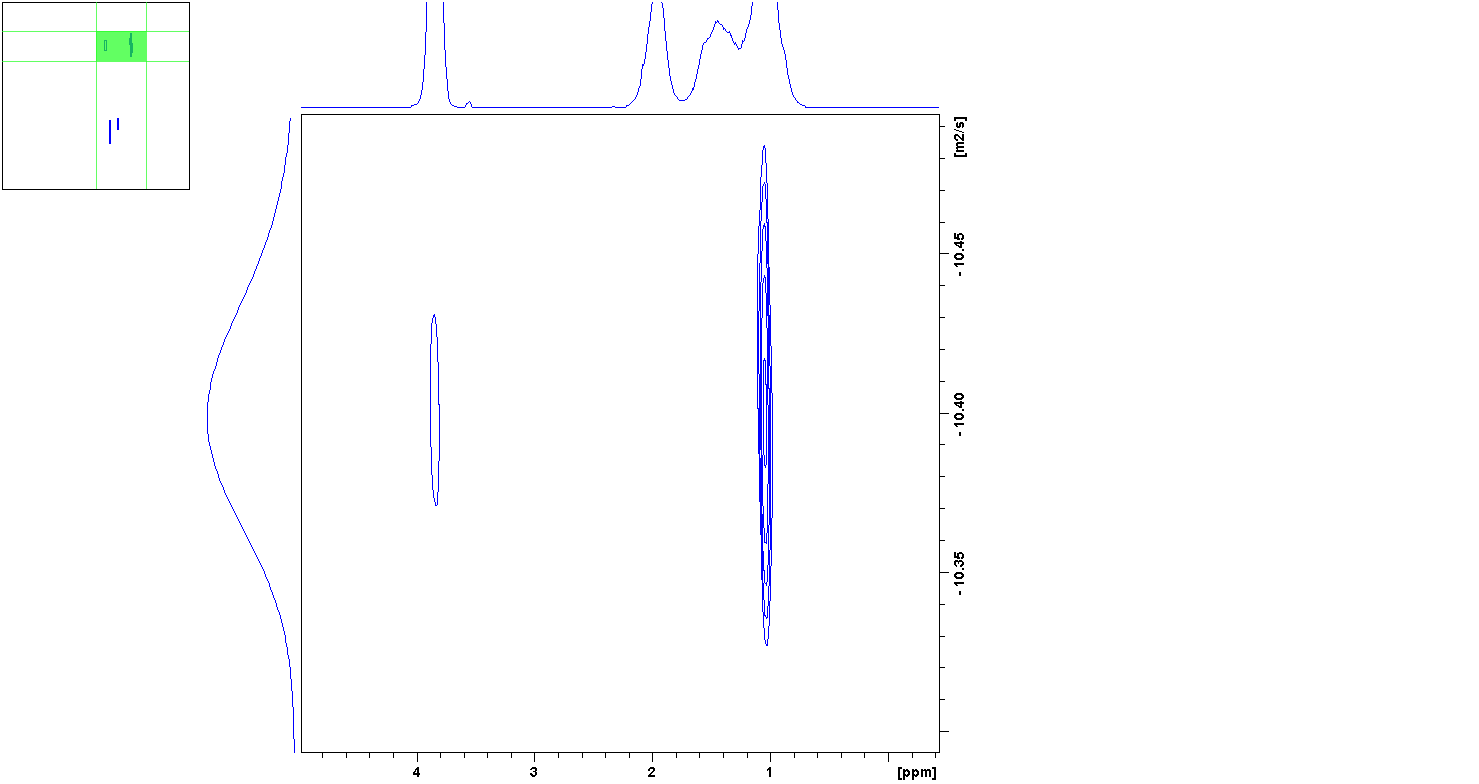 |
| Polymer 5 DOSY NMR | 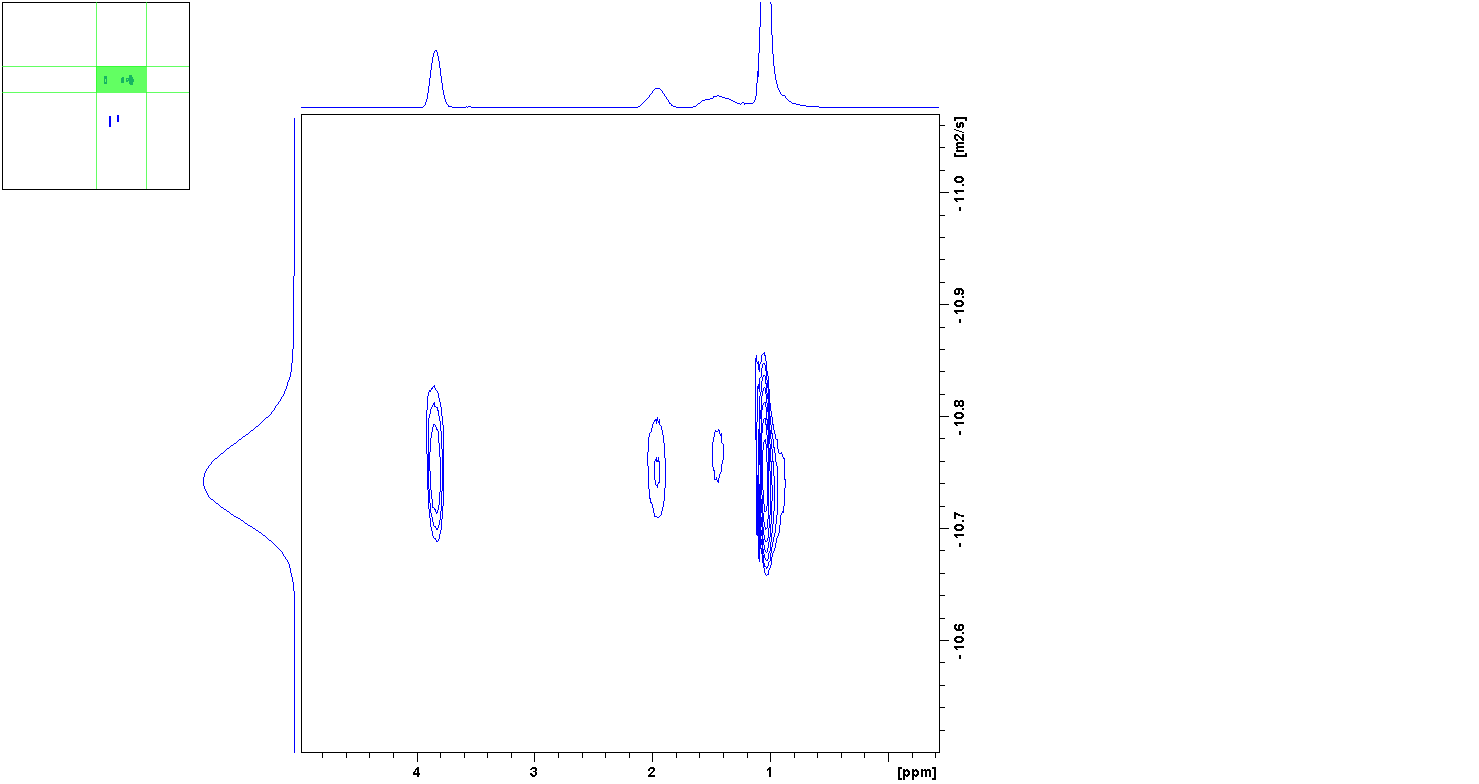 |
| Polymer 6 DOSY NMR | 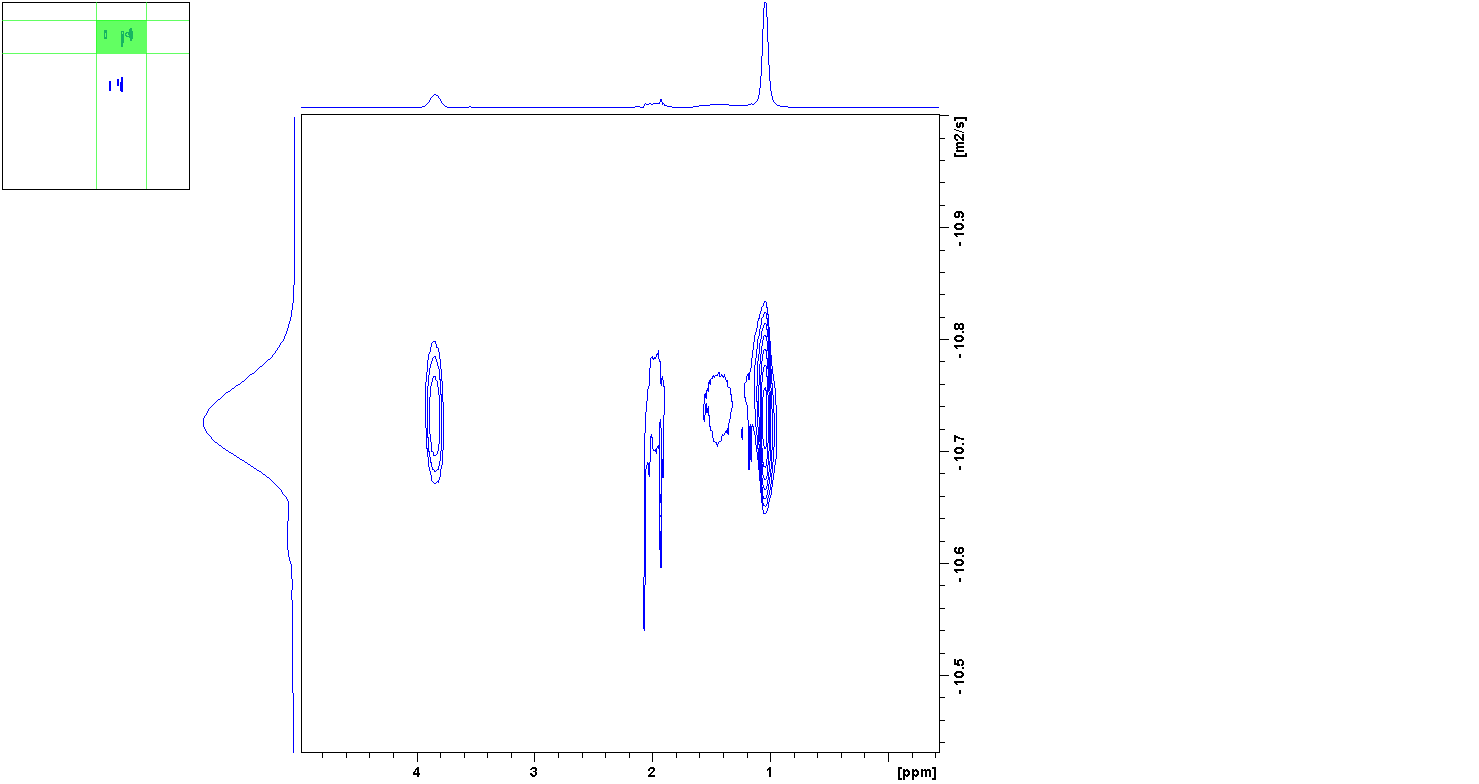 |
| Polymer 7 DOSY NMR | 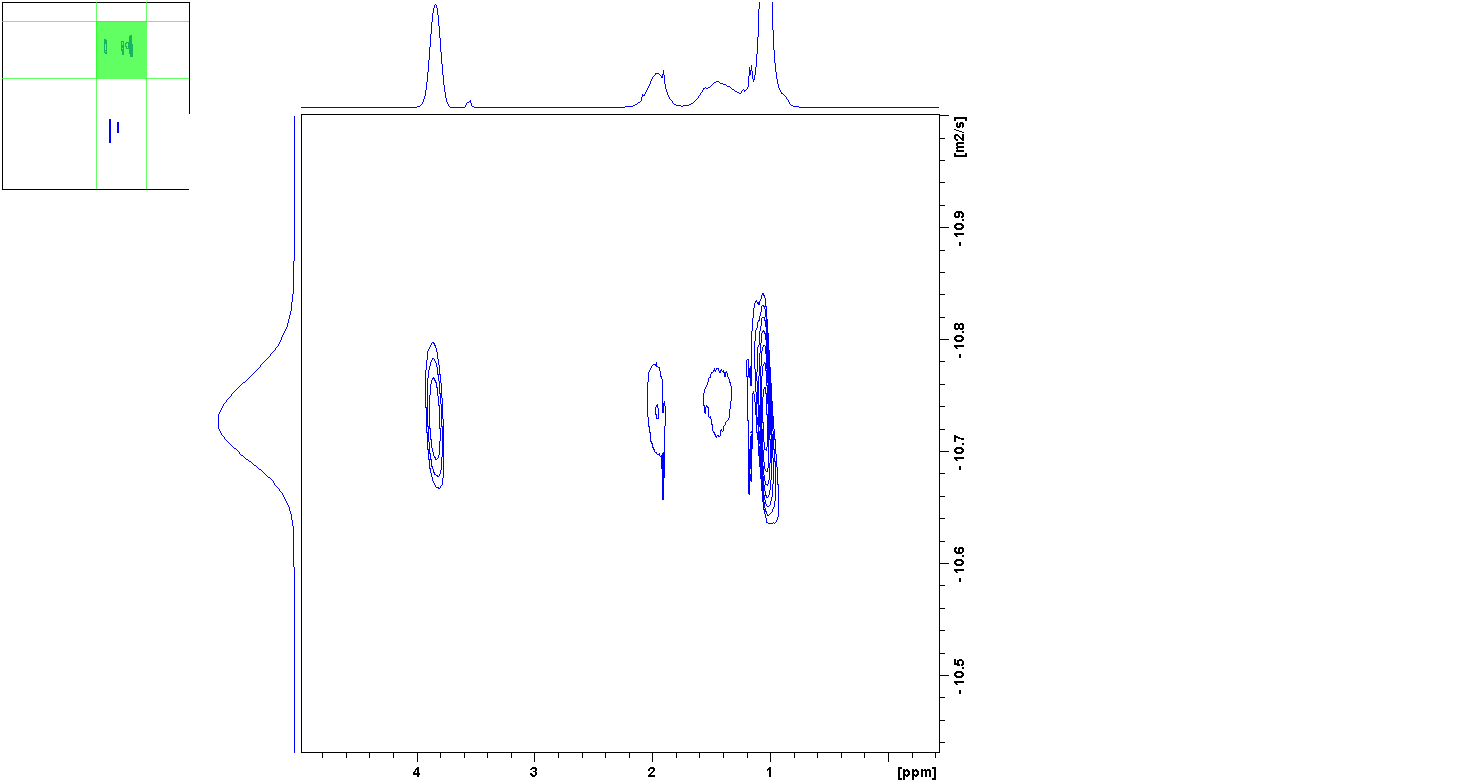 |
| Polymer 8 DOSY NMR | 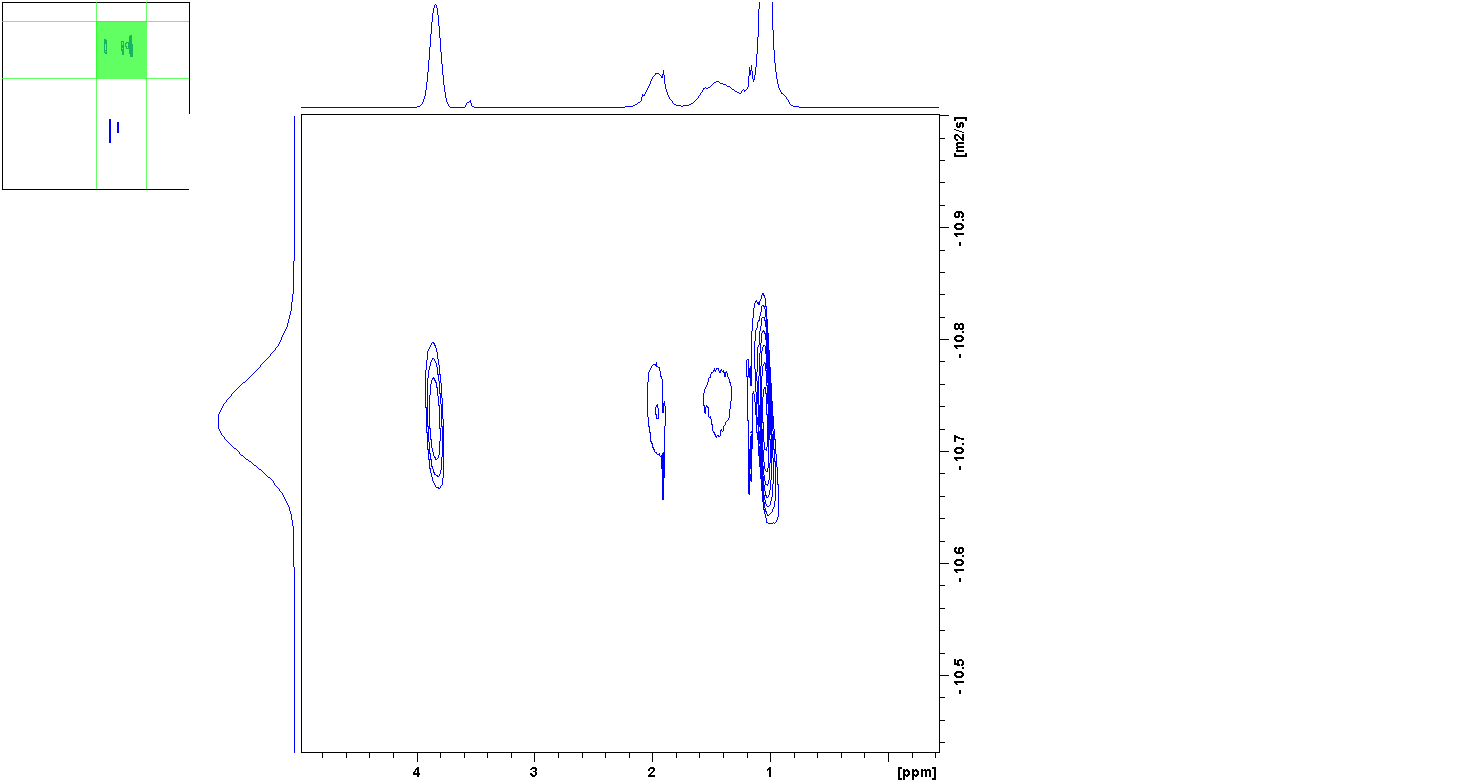 |
| Polymer 9 DOSY NMR | 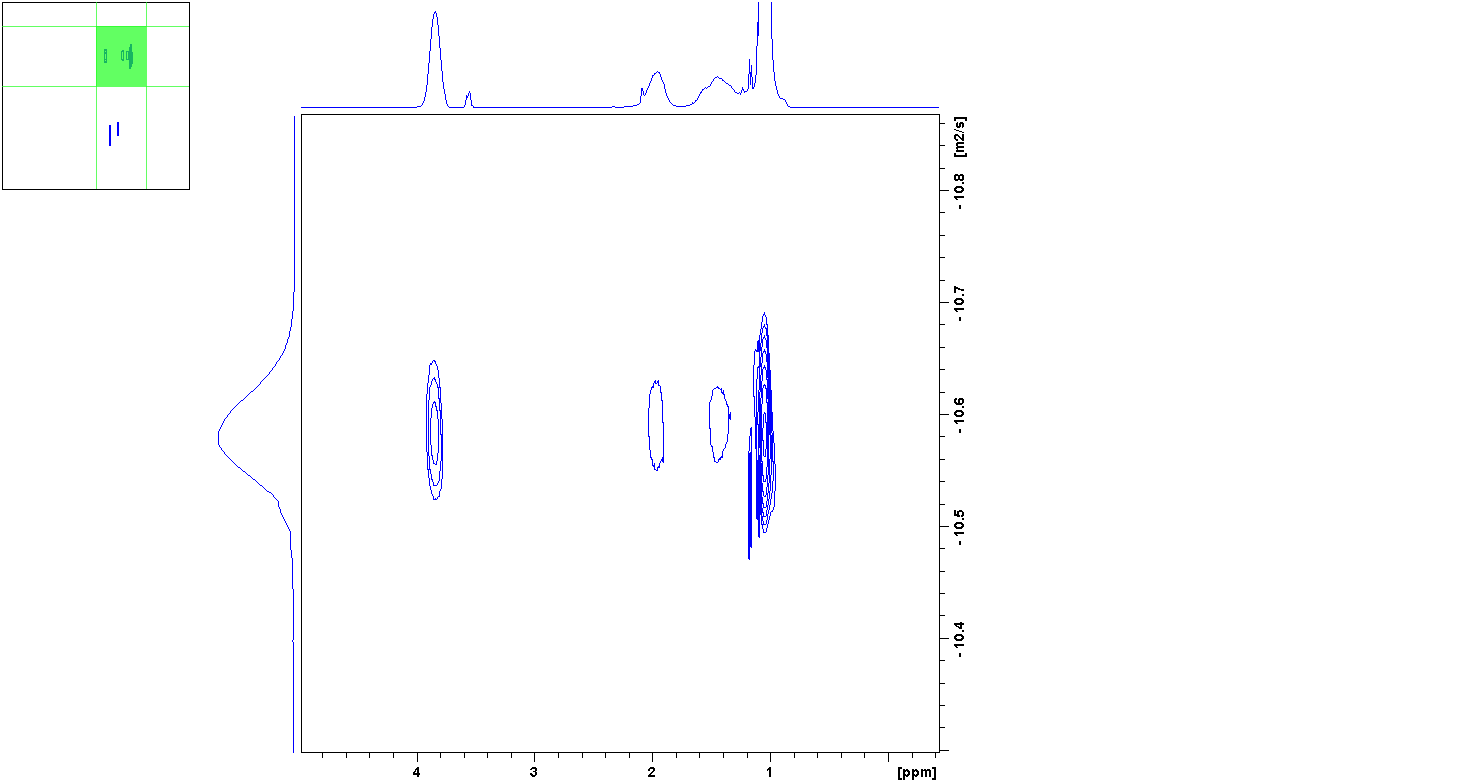 |

Figure 3 in the main article shows the retention time of PNIPAM polymers in the methanol GPC that were used to prepare the universal calibration. The full details of these are shown below in Table S9.

Table S9 – Calibration of Methanol SEC with linear PNIPAM polymers

| RT ^a^ | Log M | [ɳ] ^b^ | Measured Log (M * [ɳ]) | Calibration Log (M * [ɳ]) ^c^ |
| --- | --- | --- | --- | --- |
| 17.06 | 6.1478 | 0.107 | 5.177 | 5.239 |
| 19.41 | 5.3803 | 0.103 | 4.393 | 4.352 |
| 19.65 | 5.3775 | 0.078 | 4.269 | 4.264 |
| 20.60 | 5.0873 | 0.075 | 3.962 | 3.905 |
| 22.08 | 4.653 | 0.056 | 3.401 | 3.345 |
| 22.32 | 4.5910 | 0.046 | 3.254 | 3.257 |
| 22.67 | 4.4472 | 0.044 | 3.091 | 3.125 |
| 23.20 | 4.2672 | 0.041 | 2.880 | 2.924 |
| 23.62 | 4.1461 | 0.040 | 2.748 | 2.766 |

^a^ RT (Retention Time) in Minutes, ^b^ Intrinsic Viscosity given in dL/g ^c^ Calculated calibration trendline for [Log (M * [ɳ])]

# 7. Preparation of Linear PNIPAM using Iron based ATRP

NIPAM (2 g, 17.69 mmoles) was dissolved in DMF (4.5ml), ultra pure water (3 ml) and degassed by bubbling nitrogen through the solution.

Separately ultra pure water (6 ml), FeCl_2_ (0.1875 g, 0.943 mmoles), Me_6_Tren (0.4 ml, 1.496 mmoles) were degassed with nitrogen. 1.5 ml of this solution was added to the NIPAM solution together with MCP (50 μl) and stirred for 3 hours at room temperature under nitrogen and then precipitated in diethyl ether. It was redissolved in acetone and precipitated in diethyl ether.

# 8. Performance of poly(ethylene oxide) Standards using SEC with methanol as eluent

Poly(ethylene oxide) polymers are often used as the standards for aqueous SEC measurements. Samples that contain a mixture of three poly(ethylene oxide) samples (Agilent Easivial) of known molar masses were examined. However it was found that the PEG standards were interacting with the Agilent Polargel columns, giving low signal to noise ratios in the RI and DP response (and increased back pressure) but there were two distinct responses to the three polymers on the UV detector, one 15 – 20 minutes and the other 35 – 40 minutes.

Fig S8 – Detector response following poly(ethylene oxide) injection


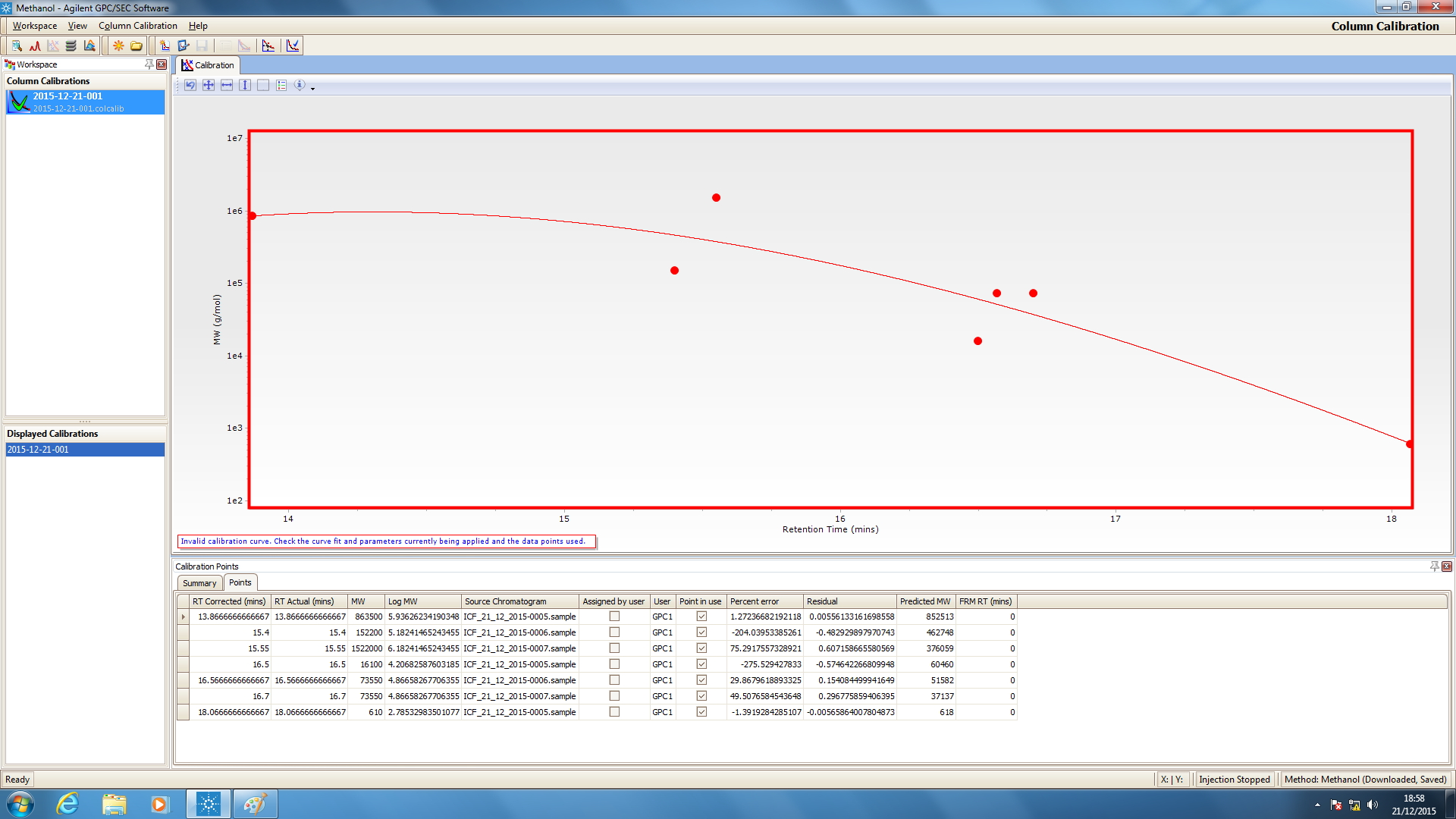


Fig S9 – Attempt to calibrate columns using PEG retention times.

The sample was flushed with water for 24 hours at 0.5 ml min^-1^ flow rate and the poly(ethylene oxide) was observed to elute from the column, although this was not recorded. Following sample extraction the column was returned to methanol. It was concluded that in methanol PEG adsorbs to PolarGel stationary phases and that standards prepared from PEG were not suitable for calibration.

# 9. Examination of the use of PolarGel Stationary Phases for SEC with THF as eluent

The flow rate was reduced to 0.2 ml min^-1^ and THF was added to the methanol solvent reservoir to give a blend containing 25 vol % of THF This was allowed to run through the chromatograph for 2 hours before adding further THF to give a blend containing 50 vol% THF. This was maintained for an additional hour before the inlet was switched to a 100 vol% THF. This was ran at 0.2 ml min^-1^ for 1 hour before the flow rate was increased to 1 ml min^-1^ for a final hour before the samples were injected.

It was noted that retention times (Mp) of the PNIPAM standards analysed in THF were equivalent to those observed when the calibration was previously carried out in methanol. This was expected following the observation from DOSY NMR that the PNIPAM has equivalent hydrodynamic radii in methanol and THF (Fig. S10). However the refractive index detector began to drift as soon as the solvent was pumped through it (Fig. S11).


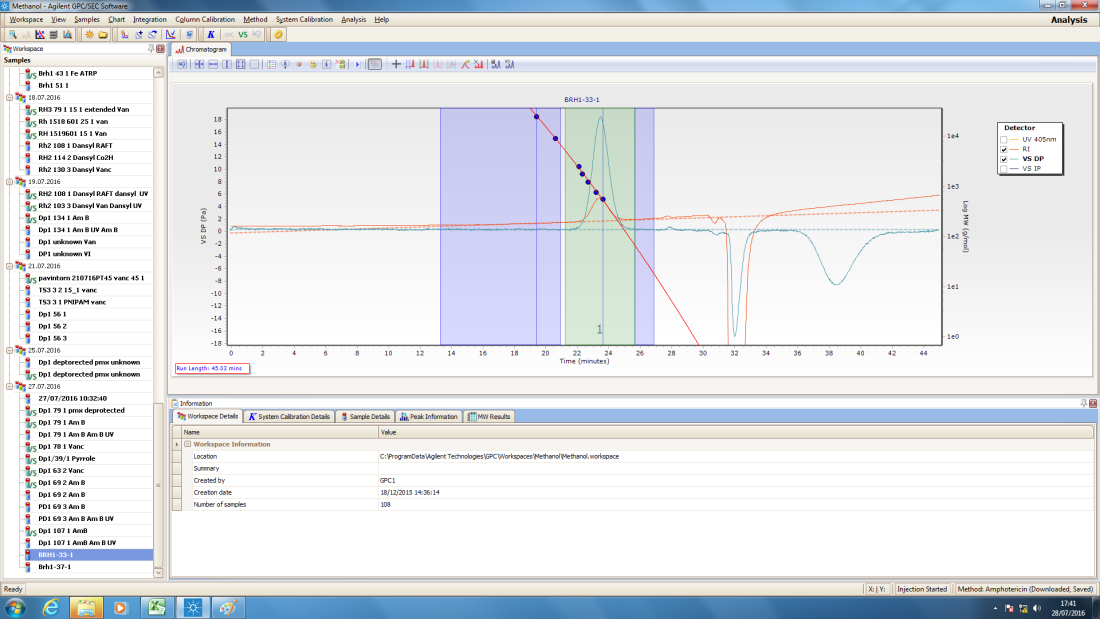


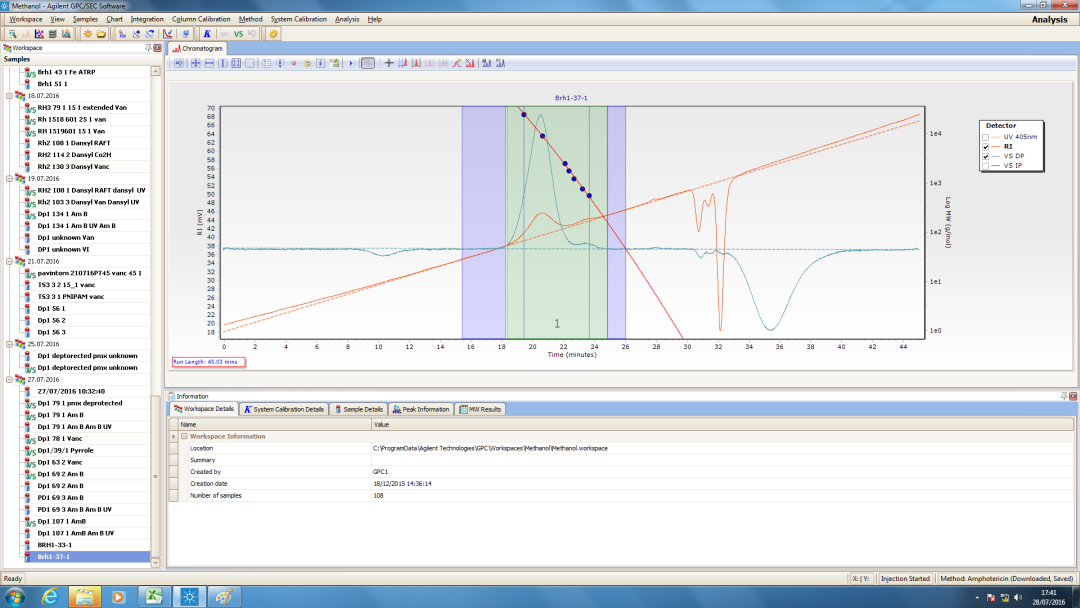


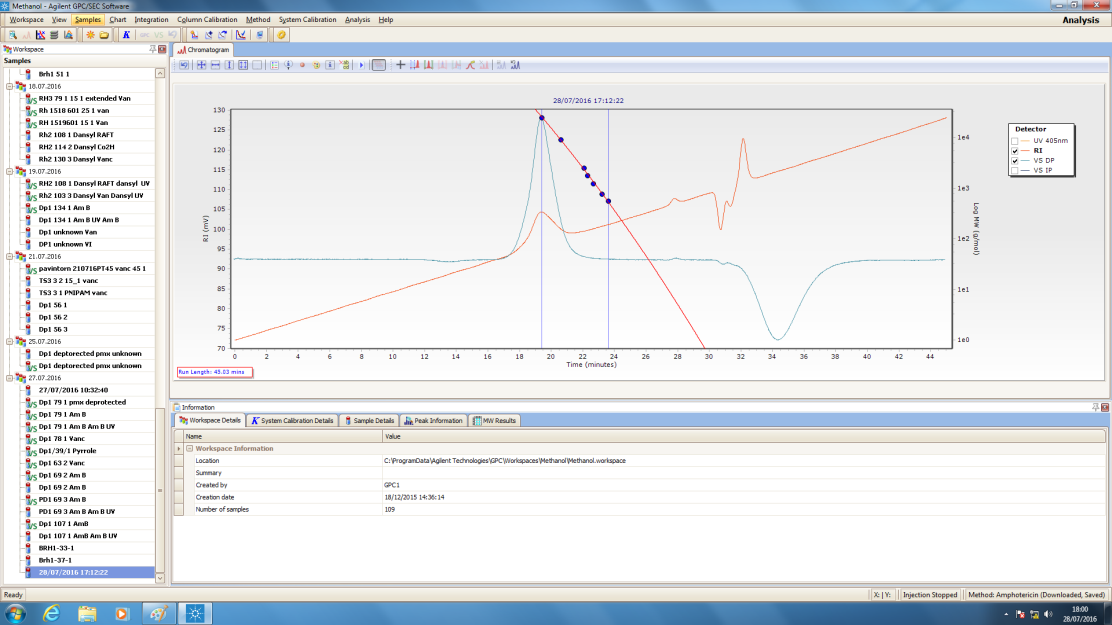


Fig S10 – Screenshots of Agilent workstation desktop showing
three example THF Raw chromatograms


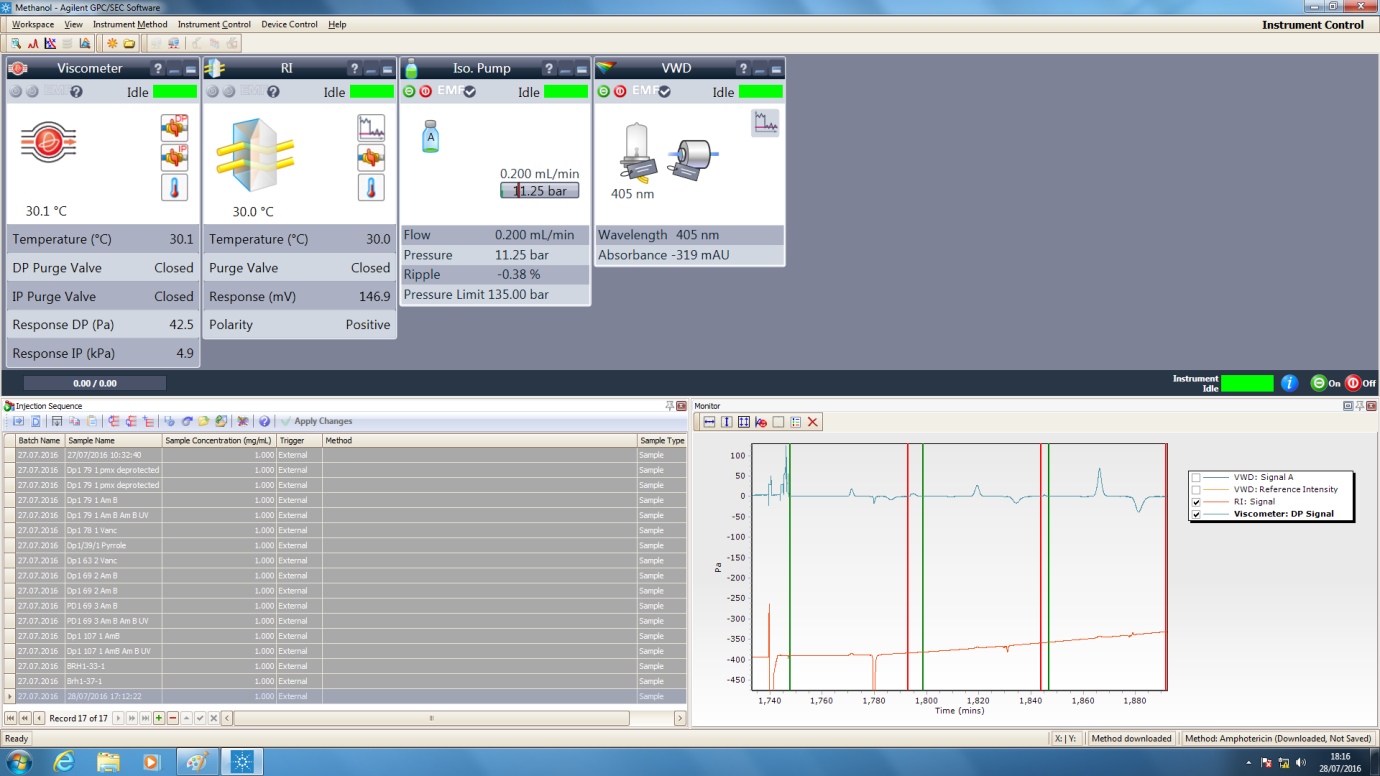


Fig. S11 – Overview of detector response through multiple injection of samples in THF solvent. Viscometer signal (top) remains constant whilst RI signal (bottom) begins to drift immediately following first sample injection and shows diminished response.

Following this the system was returned to methanol via the same system it was switched to THF and flushed with water before being recalibrated with PNIPAM standards.

# 10. Fitting alpha values across full HB-PNIPAM Molar Mass Distribution

Table S10. K, α values and goodness of fit (R^2^) of entire molar mass distribution alongside values for low molar mass component (α_1_) and high molar mas component (α_2_). The α of the full distribution is clearly inappropriately fitted and is thus not used in the main manuscript.

|  |  |  | Full Dist |  | *Component 1* | | | *Component 2* | | |
| --- | --- | --- | --- | --- | --- | --- | --- | --- | --- | --- |
| **X** : 1 | M_N_ / kg mol^-1^ | K  / dl g^-1^ | α | R^2^ | *K_1_* | α_1_ | R^2^ α_1_ | *k*_2_ | α_2_ | R^2^ α_2_ |
| 15 | 1,834.7 | 0.000002 | 5.50 | 0.90 | 0.0123 | 0.493 | 0.90 | 0.0011 | 0.948 | 0.99 |
| 25 | 1,133.9 | 0.011 | 3.33 | 0.89 | 0.045 | 0.328 | 0.86 | 0.003 | 0.785 | 0.99 |
| 35 | 555.4 | 0.0128 | 2.38 | 0.85 | 0.0504 | 0.463 | 0.88 | 0.0028 | 0.915 | 0.99 |
| 45 | 532.3 | 0.0103 | 2.55 | 0.95 | 0.2191 | 0.220 | 0.88 | 0.0132 | 0.691 | 0.99 |
| 55 | 438.8 | 0.0188 | 2.35 | 0.97 | 0.1823 | 0.307 | 0.95 | 0.0525 | 0.518 | 0.97 |
| 65 | 245.6 | 0.0137 | 2.512 | 0.98 | 0.2656 | 0.234 | 0.895 | 0.0906 | 0.431 | 0.96 |
| 75 | 412.5 | 0.0116 | 2.619 | 0.98 | 0.3103 | 0.209 | 0.885 | 0.1242 | 0.391 | 0.95 |
| 85 | 624.6 | 0.0147 | 2.492 | 0.98 | 0.1952 | 0.303 | 0.925 | 0.1226 | 0.390 | 0.96 |

11. ^1^H DOSY and SEC of HB-PNIPAM in MeOD

Table S11 – Hydrodynamic Radii of HB-PNIPAM polymers from Methanol SEC (Fig. 6) and DOSY (Fig. 8).

|  | SEC | | | DOSY | | | | |
| --- | --- | --- | --- | --- | --- | --- | --- | --- |
| NIPAM: VBP^a^ | R_Hp_ | R_Hn_ | R_Hw_ | -logD (S) | -logD (P) | R_Hp_ | R_Hn_ | R_Hw_ |
| MeOD | - | - | - | 9.087 | - | - | - | - |
| 15:1 | 26.6 | 19.7 | 26.38 | 8.616 | 10.224 | 19.9 | 23.6 | 24.5 |
| 25:1 | 28.02 | 17.89 | 27.40 | 8.648 | 10.243 | 19.3 | 23.6 | 24.5 |
| 35:1 | 29.59 | 16.29 | 27.08 | 8.636 | 10.208 | 18.3 | 22.9 | 24.0 |
| 45:1 | 27.36 | 15.71 | 24.89 | 8.625 | 10.182 | 17.7 | 20.6 | 21.3 |
| 55:1 | 26.88 | 16.02 | 24.04 | 8.641 | 10.207 | 18.1 | 20.4 | 20.9 |
| 65:1 | 26.39 | 15.33 | 23.39 | 8.663 | 10.215 | 17.5 | 19.8 | 20.4 |
| 75:1 | 26.75 | 16.05 | 23.41 | 8.636 | 10.212 | 18.5 | 19.9 | 20.6 |
| 85:1 | 27.09 | 16.93 | 23.89 | 8.639 | 10.211 | 18.3 | 20.4 | 21.0 |

Branched polymers analysed by GPC were also analysed by DOSY NMR as shown in Table 4. In this the R_Hp_, R_HN_, R_HW_ from SEC measurement were compared with the R_Hp_ from DOSY analysis. This is shown graphically below in Fig. S13.

Fig S13 – Hydrodynamic radii trends depending on method used.

This R_HP_ DOSY peak was generated using the peak of the raw ^1^H proton diffusion distribution whilst R_Hn_ and R_Hw_ come from data shown in Fig. 8.

12. Column Retention Time (Toluene Injection)

The retention time of the Polargel columns was determined by injection of 10 μl Toluene / 2 ml Methanol flow rate markers through the analytical system with the columns attached, and removed from, the mobile phase flow. The data is shown in Fig. S14 (separate) and S15 (overlaid) and shows that the solvent passed through the columns after 33.712 minutes (std. dev. 0.032 minutes across three repeats) and in the absence of columns reached the detectors 0.5333 minutes (0.041 minutes std. dev across 3 repeats) after injection. The hold up volume, VH, of the column set indicates the total volume of the internal pores and the interstitial volume. VH is given by equation 1:

VH = (T_R_ – T_nc_) x F Equation ESI1

T_R_ = retention time, T_nc_ = retention time without the columns, F = Flow rate

Fig S15 – Detector Response from Toluene injection through Methanol GPC with (right) and without (left) Polargel columns present.

Fig. S16 – Overlaid detector responses following toluene injection through Methanol GPC.

Together this showed the column retention time for toluene was 33.1787 minutes.

13. High Molar Mass Excluded Samples

During this study several high molar mass PNIPAM polymers were prepared which were too large to analyse using THF GPC (they were excluded from the column). These samples were also injected into the Methanol GPC system to observe the retention time of excluded polymer samples (Fig. S17).

Despite being synthesised using different initiator:monomer ratios they eluted at similar retention times, indicating high molar mass exclusion. The average retention time of these polymers (T_PNIPAM_)was 13.97 minutes, with a std. deviation of 0.036 minutes. During this study no polymers showed any retention before 14 minutes indicating that this is a suitable system for study of high molar mass systems.

The retention volume, V_PNIPAM,_ (= (T_PNIPAM_ – T_nc_) x F) is equal to the interstitial volume so that the volume of the internal pores, V_pores_ is:

V_pores_ = VH - V_PNIPAM_ Equation ESI2

Fig. S17 –Detector Response of 3 large molar mass PNIPAM polymers excluded from Polargel columns

14. System Calibration Reports

1. Example THF Calibration Report


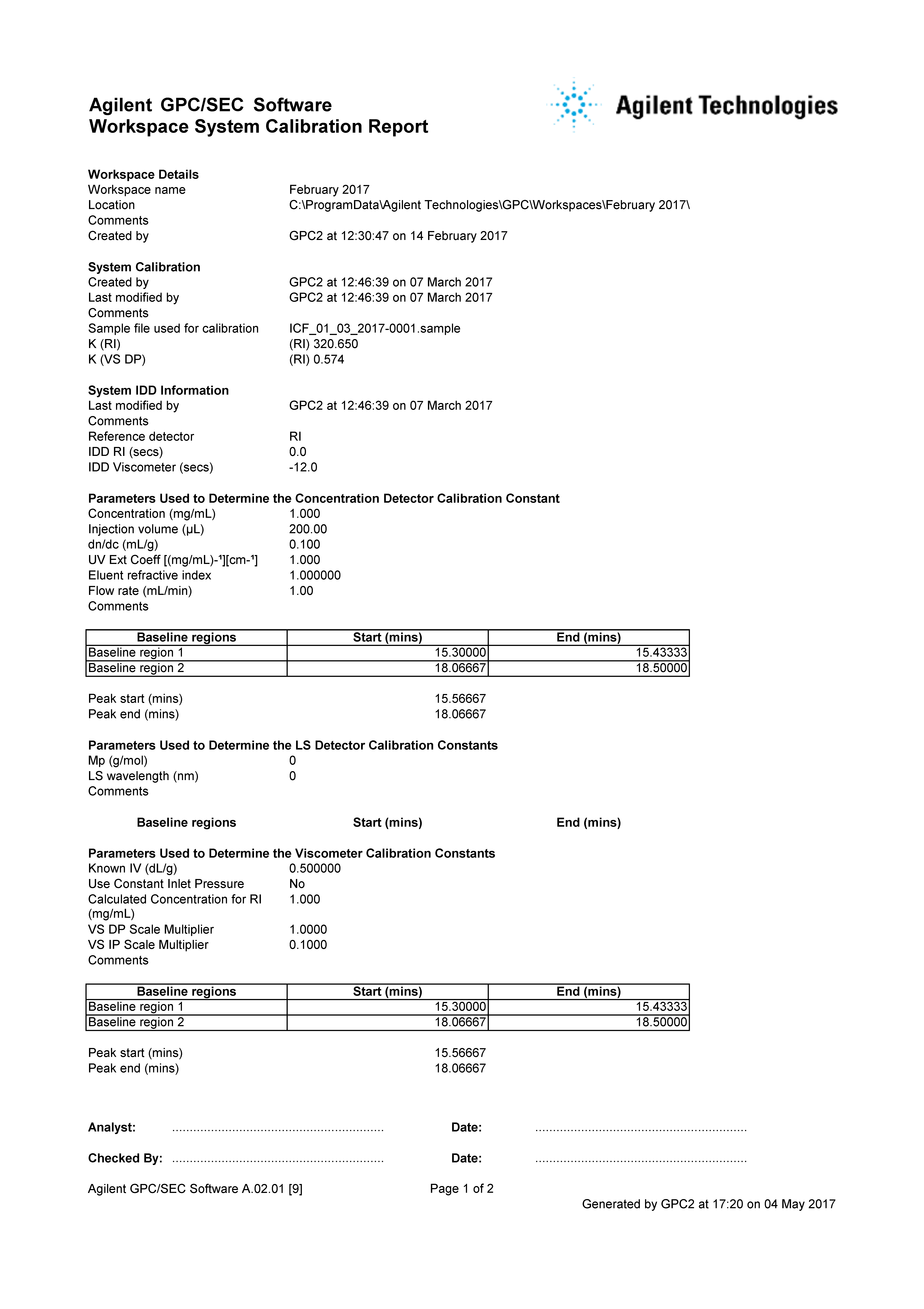


b) Methanol GPC System Calibration Report


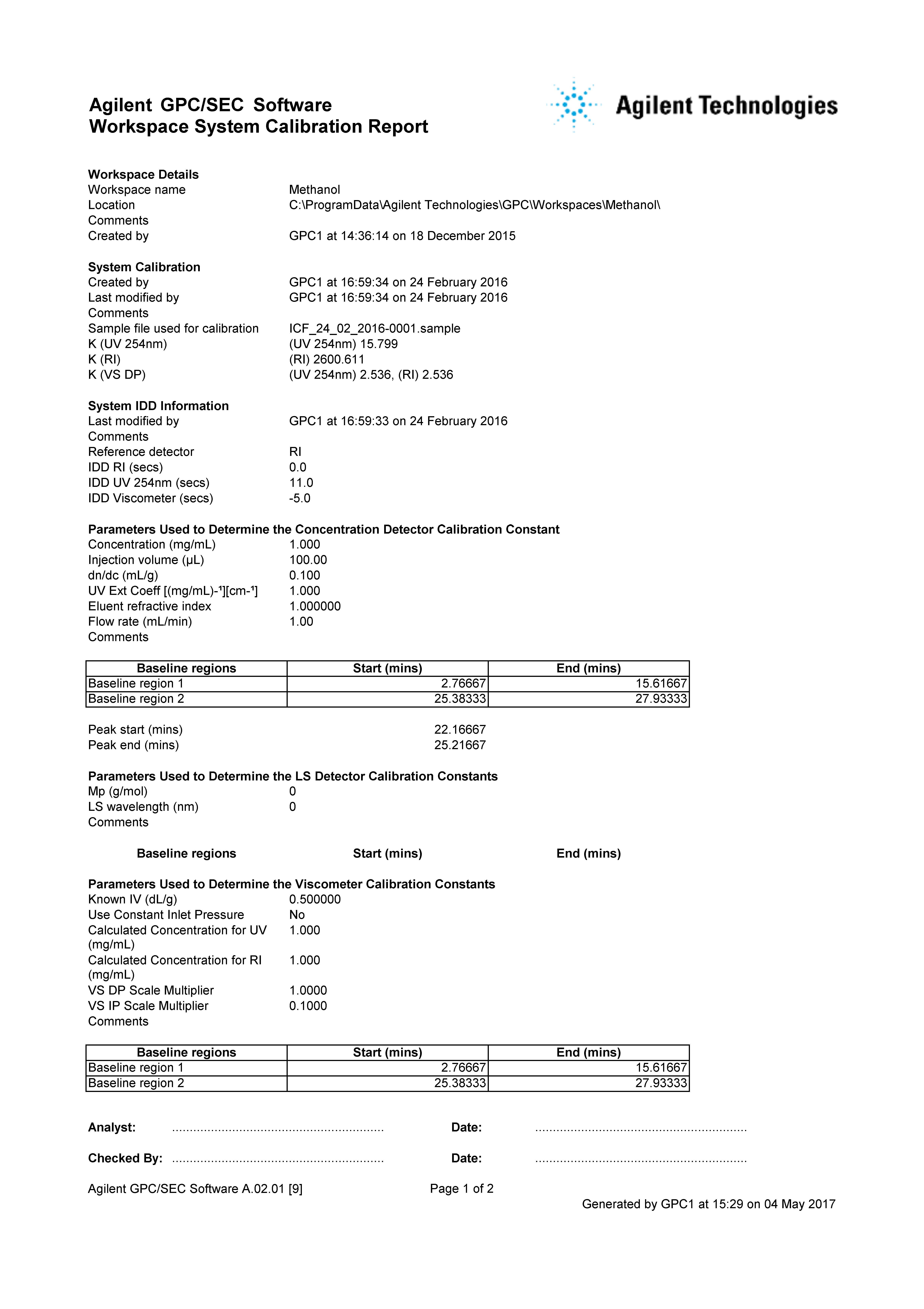


15. Supporting Information References

1. Swan, I.; Reid, M.; Howe, P. W. A.; Connell, M. A.; Nilsson, M.; Moore, M. A.; Morris, G. A., Sample convection in liquid-state NMR: Why it is always with us, and what we can do about it. *Journal of Magnetic Resonance* **2015,** *252*, 120-129.

2. Wang, S. T.; Hansen, P. M. T.; Barringer, S. A., Effects of sucrose and CMC on D2O diffusion in water. *Food Hydrocolloids* **1998,** *12* (2), 115-119.

3. Mills, R., Self-diffusion in normal and heavy water in the range 1-45.deg. *The Journal of Physical Chemistry* **1973,** *77* (5), 685-688.

4. Amu, T. C., The unperturbed molecular dimensions of poly(ethylene oxide) in aqueous solutions from intrinsic viscosity measurements and the evaluation of the theta temperature. *Polymer* **1982,** *23* (12), 1775-1779.

5. Kawaguchi, S.; Imai, G.; Suzuki, J.; Miyahara, A.; Kitano, T.; Ito, K., Aqueous solution properties of oligo- and poly(ethylene oxide) by static light scattering and intrinsic viscosity. *Polymer* **1997,** *38* (12), 2885-2891.

6. Kawaguchi, M.; Mikura, M.; Takahashi, A., Hydrodynamic studies on adsorption of poly(ethylene oxide) in porous media. 2. Molecular weight dependence of hydrodynamic thickness. *Macromolecules* **1984,** *17* (10), 2063-2065.

7. Armstrong, J. K.; Wenby, R. B.; Meiselman, H. J.; Fisher, T. C., The Hydrodynamic Radii of Macromolecules and Their Effect on Red Blood Cell Aggregation. *Biophysical Journal* **2004,** *87* (6), 4259-4270.

8. Woodley, D. M.; Dam, C.; Lam, H.; LeCave, M.; Devanand, K.; Selser, J. C., Draining and long-ranged interactions in the poly(ethylene oxide)/water good solvent system. *Macromolecules* **1992,** *25* (20), 5283-5286.

9. Kirinčič, S.; Klofutar, C., Viscosity of aqueous solutions of poly(ethylene glycol)s at 298.15 K. *Fluid Phase Equilibria* **1999,** *155* (2), 311-325.

10. Fabula, A. G., On the relevance of intrinsic viscosity to the concentration dependence of the toms effect. *Journal of Polymer Science Part A: General Papers* **1965,** *3* (10), 3662-3663.
